# PIRCHE-II scores prove useful as a predictive biomarker among kidney transplant recipients with rejection: An analysis of indication and follow-up biopsies

**DOI:** 10.3389/fimmu.2022.949933

**Published:** 2022-08-17

**Authors:** Tahm Spitznagel, Laurenz S. Matter, Yves L. Kaufmann, Jakob Nilsson, Seraina von Moos, Thomas Schachtner

**Affiliations:** ^1^ Division of Nephrology, University Hospital of Zurich (USZ), Zurich, Switzerland; ^2^ Division of Immunology, University Hospital of Zurich (USZ), Zurich, Switzerland

**Keywords:** HLA epitope mismatch, TCMR, ABMR, borderline rejection, kidney allograft biopsy

## Abstract

**Background:**

Indication biopsies for deterioration of kidney allograft function often require follow-up biopsies to assess treatment response or lack of improvement. Immune-mediated injury, namely borderline rejection (BLR), T-cell mediated rejection (TCMR), or antibody-mediated rejection (ABMR), results from preformed or *de novo* alloreactivity due to donor and recipient HLA-mismatches. The impact of HLA-mismatches on alloreactivity is determined by highly immunogenic HLA-epitopes.

**Methods:**

We analyzed 123 kidney transplant recipients (KTRs) from 2009 to 2019 who underwent a first indication and a follow-up biopsy. KTRs were divided into three groups according to the first biopsy: No rejection (NR)/BLR (n=68); TCMR (n=21); ABMR (n=34). The HLA-derived epitope-mismatches were calculated using the Predicted Indirectly Recognizable HLA-Epitopes (PIRCHE-II) algorithm.

**Results:**

Group NR/BLR: KTRs with higher total PIRCHE-II scores were more likely to develop TCMR in the follow-up biopsy (p=0.031). Interestingly, these differences were significant for both HLA-class I- (p=0.017) and HLA-class II-derived (p=0.017) PIRCHE-II scores. Group TCMR: KTRs with ongoing TCMR in the follow-up biopsy were more likely to show higher total PIRCHE-II scores (median 101.50 vs. 74.00). Group ABMR: KTRs with higher total PIRCHE-II scores were more likely to show an increase in the microvascular inflammation score in the follow-up biopsy. This difference was more pronounced for the HLA-class II-derived PIRCHE-II scores (median 70.00 vs. 31.76; p=0.086).

**Conclusions:**

PIRCHE-II scores may prove useful as a biomarker to predict the histopathological changes of immune-related injury from a first indication to a follow-up biopsy. This immunological risk stratification may contribute to individualized treatment strategies.

## Introduction

Kidney allograft rejection due to immune-mediated injury remains a common complication after kidney transplantation, partly due to the increasing number of re-transplantations and transplantation of otherwise sensitized kidney transplant recipients (KTRs). Immune-mediated injury can be subdivided into T-cell-mediated rejection (TCMR) and antibody-mediated rejection (ABMR). In contrast, borderline rejection (BLR) contains various histologic lesions, ranging from mild inflammation to clinically significant TCMR (1, 2).he Banff classification was developed to objectify the results from kidney biopsies (3). This classification estimates the presence and severity of histopathological changes in the different compartments of the kidney (4). The Banff classification has been modified several times over the last years, and associated with this, the diagnostic criteria for BLR and ABMR have changed (5–9).

Recently, the Predicted Indirectly Recognizable HLA Epitopes (PIRCHE-II) algorithm (10, 11) was developed to predict T-cell-related immune responses against donor HLA-derived peptides. Considering an electrostatic mismatch algorithm, PIRCHE-II scores go beyond a simple amino acid sequence comparison and aim to discriminate immunogenicity (12–15). The PIRCHE-II scores - as a marker for the allo-immunogenicity of donor-recipient HLA-mismatch – were associated with the risk for developing *de novo* donor-specific antibodies (DSA) and long-term kidney allograft survival in two large kidney transplant cohorts (12, 16). Lachmann et al. demonstrated in a cohort of 2787 kidney transplants that high PIRCHE-II scores are a strong predictor of the development of *de novo* DSA (16). Recently for the first time, Geneugelijk et al. and Senev et al. revealed that a high PIRCHE-II score is associated with an increased risk of TCMR and kidney allograft failure (17, 18). Antibody patterns of highly-sensitized KTRs indicated that only a small number of mismatched HLA-epitopes induce antibody formation (11, 19–23). Identifying these potentially immunogenic epitopes on HLA antigens may potentially discriminate immunogenicity in a more detailed way than measuring the number of HLA mismatches. A high number of HLA-epitope mismatches translates into a higher risk that one of the mismatched HLA-epitopes is highly immunogenic, which can facilitate the development of *de novo* DSA (24).

The PIRCHE-II algorithm calculates the number of theoretical HLA-epitopes consisting of 9 amino acids capable of causing an indirect alloreactive response that involves CD4+ T-cell recognition of HLA class-II presented donor HLA-peptides (10, 25). These activated donor-reactive CD4+ T-cells themselves can then subsequently support the development of *de novo DSA* by providing T-cell help to donor-HLA-reactive B-cells (16).

In case of clinical suspicion of kidney allograft rejection, the diagnosis must be confirmed by an indication biopsy and classified according to the Banff criteria into BLR, TCMR, and ABMR. In general, there are two main reasons for an indication biopsy, either worsening kidney allograft function or the development of proteinuria in the presence or absence of DSA (26–31). It is not uncommon for some KTRs to require a follow-up biopsy due to an ambiguous clinical course. A follow-up biopsy is mainly indicated for one of the following three reasons: (1) If the histological findings in an indication biopsy show no rejection (NR) or BLR, but the clinical course is still suspicious of kidney allograft rejection, (2) if a KTR was treated for TCMR, but an unsatisfactory clinical course urges the clinician to investigate the histological response to therapy, or (3) if a KTR is diagnosed with ABMR in a first indication biopsy and the progression of microvascular inflammation is to be assessed either after treatment or after adjustment of maintenance immunosuppression.

The current study applies PIRCHE-II socres as a predictive biomarker for the progression of immune-mediated kidney allograft injury: (1) Does the PIRCHE-II score predict acute rejection (TCMR and ABMR) in follow-up biopsies in KTRs with NR/BLR in a first indication biopsy? (2) Does PIRCHE-II predict recovery of histological findings in follow-up biopsies in KTRs with TCMR in a first indication biopsy? (3) Does PIRCHE-II predict the severity of microvascular inflammation in follow-up biopsies in KTRs with ABMR in a first indication biopsy?

## Patients and methods

### Patients

Our study was approved by the “Cantonal Ethics Commission Review Board of Zurich,” Switzerland (KEK-ZH Number 2020-02817) and has been conducted in compliance with the declaration of Helsinki.

**Figure 1:**
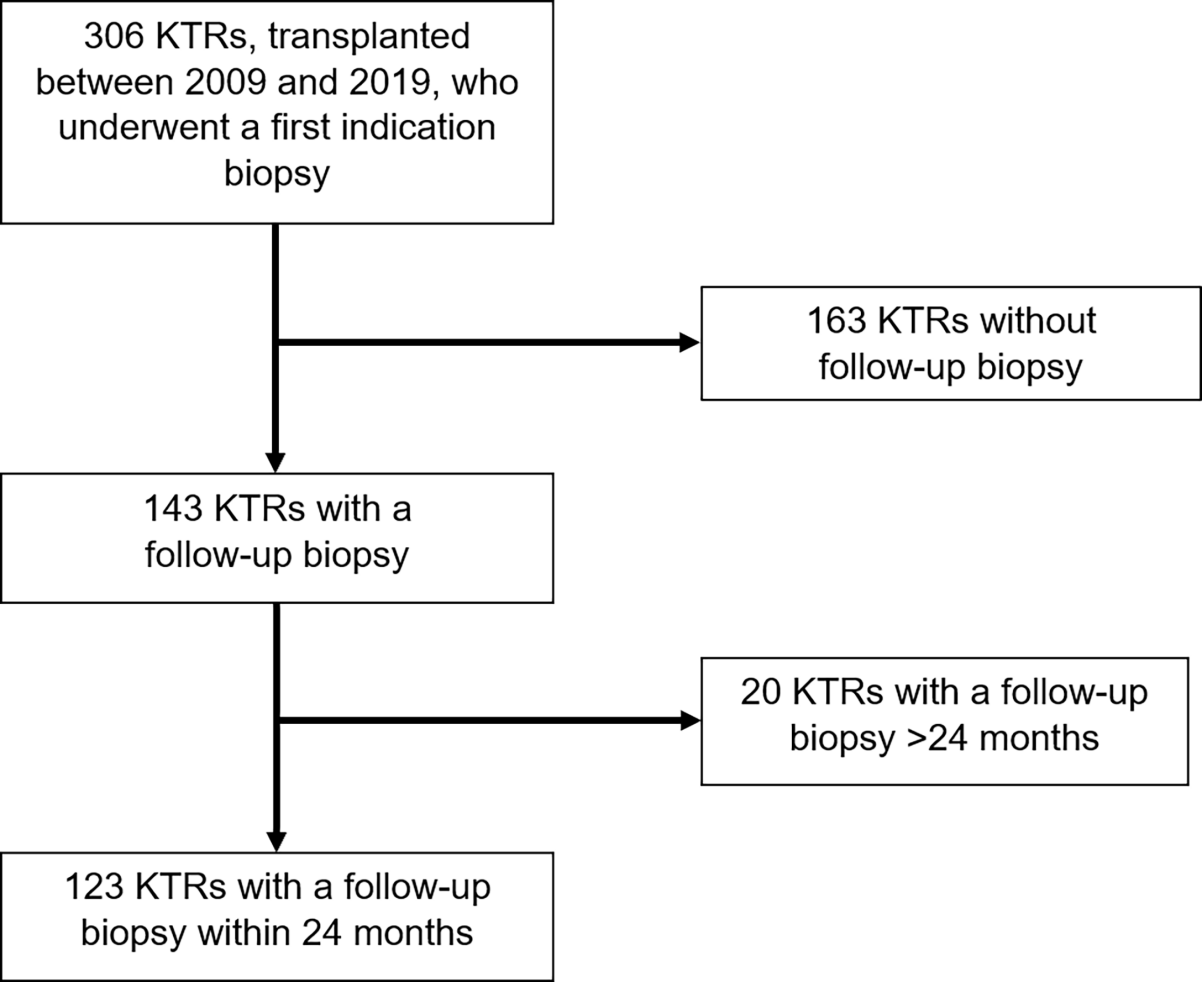
Patient inclusion and exclusion algorithm.

We performed an observational study of 306 KTRs who underwent kidney transplantation at the University Hospital of Zurich between January 1, 2009, and December 30, 2019. They all received an indication biopsy, either because of a deterioration in kidney allograft function or proteinuria. A follow-up biopsy was performed on 143 KTRs. From this cohort, we selected 123 KTRs, who received their follow-up biopsy within 24 months after the indication biopsy. All KTRs had a minimum follow-up period of one year (**Figure 1**).

KTRs were divided into three groups: (1) Group NR/BLR included 68 KTRs, who showed histological NR/BLR in the first indication biopsy and NR/BLR, TCMR, or ABMR in the follow-up biopsy; (2) Group TCMR included 21 KTRs, who showed histological TCMR in the first indication biopsy and either NR/BLR or ongoing TCMR or ABMR in the follow-up biopsy; (3) Group ABMR included 34 KTRs, who showed histological ABMR in the first indication biopsy and then either NR/BLR, TCMR or ABMR in the follow-up biopsy.

Post-transplant care was carried out according to a standardized clinical protocol with appointments in the outpatient clinic twice a week in weeks 2 and 3, at weeks 4, 5, 6, 8, 10, and 12, and at months 4, 5, 6, 8, 10 and 12, with at least 16 visits within the first year after transplantation. Subsequently, quarterly check-ups were performed with a local nephrologist in conjunction with at least annual follow-up visits in our outpatient clinic. The anti-HLA antibody testing was performed using a Luminex-based assay, LABScreen Mix, or LABScreen Single Antigen (One Lambda, Canoga Park, CA, USA) on the day of transplantation and in months 3, 6, 12, and annually after that, additional testing was performed in the case of unexpected graft dysfunction.

### Induction and maintenance immunosuppression

The choice of induction therapy was based on immunological risk. KTRs with a low-immunologic risk received IL-2-receptor blockade with basiliximab. KTRs with a high-immunologic risk received lymphocyte-depleting induction with thymoglobulin. ABO desensitization included a single dose of rituximab and blood group-specific immunoadsorption before transplantation. The primary immunosuppression consisted of a triple-drug combination of a calcineurin inhibitor (CNI), tacrolimus or cyclosporine, antimetabolite (mycophenolate mofetil (MMF) or mycophenolic acid (MPA) or azathioprine), and steroids. The initial dose of tacrolimus was 0.2 mg/kg body weight/day, and trough levels were maintained at 10-15 µg/l until week 6, at 8-12 µg/l until week 12, at 7-10 µg/l until month 12, at 6-8 µg/l until month 24, and at 4-6 µg/l after that. The initial dose of cyclosporine was 8 mg/kg body weight, and target trough levels were at 200-250 µg/l until week 6, at 180-220 µg/l until week 12, at 150-200 µg/l until month 12, at 80-120 µg/l until month 24, and at 60-100 µg/l after that. The dosage of MMF was 2000 mg/day, and the dosage of MPA was 1440 mg/day. Steroid tapering was performed over 12 weeks to a dose of 5 mg prednisone/day. According to immunologic risk, steroid withdrawal was implemented.

### Assessment of kidney allograft function and kidney allograft biopsies

To evaluate kidney allograft function and proteinuria, 5 time periods were evaluated: (1)12 months before the indication biopsy; (2) at the time of the indication biopsy; (3)12 months before the follow-up biopsy; (4)at the time of the follow-up biopsy; (5)12 months after the follow-up biopsy. - Creatinine and proteinuria baselines over 12 months were calculated using the three lowest values from each period to form the average. Creatinine and proteinuria baselines two and four were formed using the value of the biopsy day.

In total, 123 KTRs with at least one follow-up biopsy were included in the analysis. The biopsies were evaluated by an experienced renal pathologist and were not blinded to clinical information. The rejection was classified according to the Banff 2018 reference guide (1).

### Calculation of predicted indirectly recognizable HLA-Epitopes (PIRCHE-II) scores

The HLA-derived mismatched peptide epitopes presented by KTRs HLA-molecules were calculated using the PIRCHE-II algorithm. Presentation of both HLA class I (HLA-A, B, C) and HLA class II derived peptides (HLA-DR, DQ) were calculated for each HLA locus, and designated PIRCHE-II-A, B, C, DR, and DQ. The total PIRCHE-II score is the sum of PIRCHE-II-1 and PIRCHE-II-2. PIRCHE-II-1 is composed of PIRCHE-II-A, PIRCHE-II-B and PIRCHE-II-C. PIRCHE-II-2 is composed of PIRCHE-II-DR and PIRCHE-II-DQ. Detection of HLA antigens was performed by DNA-based HLA-typing technology using blood samples. Either sequence-specific oligonucleotide (SSO) or sequence-specific primer (SSP) technologies were used to generate low-resolution HLA typing results. The imputation of probable allele resolution results needed for the PIRCHE-II calculation was achieved by the use of the imputation algorithm included in the PIRCHE-II calculation. The PIRCHE-II algorithm is available online (https://www.PIRCHE-II.org).

### Statistical methods

Statistical analysis was performed using IBM SPSS Version 27 (SPSS, Chicago, IL, USA). For comparisons of study groups, Mann–Whitney U-Test was used for nonparametric independent samples. A two-sided Wilcoxon signed-rank test for nonparametric dependent samples was used to compare paired samples. Outcomes were measured with Kaplan-Meier models, and log-rank tests measured overall strata comparisons. Using Fisher’s exact test for categorical variables, clinical characteristics were compared across groups. Boxplots show median, interquartile range (IQR), and 95^th^ percentile.

## Results

### Discrepancy between HLA mismatches and molecular HLA-epitope mismatches

**Figure 2:**
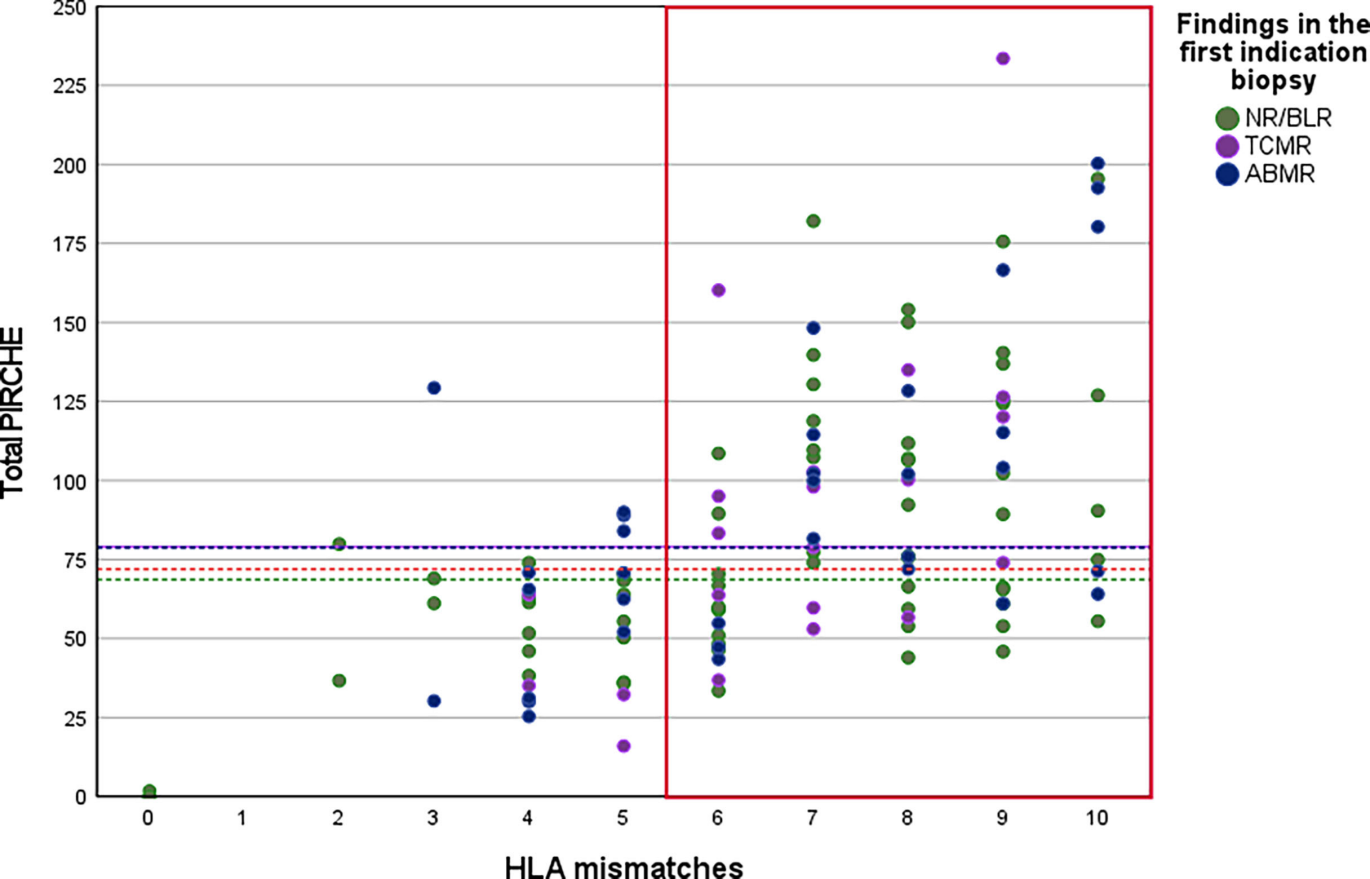
Distribution of total PIRCHE-II scores compared to total HLA-mismatches. PIRCHE-II scores and the number of HLA mismatches were calculated from HLA class I (HLA-A, B, C) and HLA class II (HLA-DR, DQ) mismatches. Median PIRCHE-II scores for Group NR/BLR, Group TCMR, and Group ABMR were 68.66, 79.03, and 79.99, respectively.

The median total PIRCHE-II score of all KTRs was 71.96 (range 0.00-233.55). 34.88% of those KTRs with an HLA mismatch of six or higher had a total PIRCHE-II score lower than the median total PIRCHE-II score. Only 13.51% of those KTRs with an HLA mismatch of five or less had a total PIRCHE-II score higher than the median total PIRCHE-II score. **Figure 2** shows the distribution of the total PIRCHE-II score concerning the number of HLA mismatches.

### Overall patient characteristics

The basic characteristics and the clinical and biopsy-related data are shown in **Tables 1**–**3** and **Supplement Tables 1–3**.

**Table 1:** Basic characteristics of 68 KTRs with NR/BLR in the first indication biopsy and grouping based on the results of the follow-up biopsy.

	Total	NR/BLR	ABMR	TCMR	*P*
	n=68	n=42	n=9	n=17	*NR/BLR vs. TCMR*
Recipient characteristics					
Recipient age, years*	54.5 (18-75)	54.5 (18-75)	52 (30-66)	58 (26-69)	*0.315*
Recipient, male sex, n (%)	36 (53)	19 (45)	7 (78)	10 (59)	*0.399*
Living donation, n (%)	21 (31)	14 (33)	2 (22)	5 (29)	*1.000*
Deceased donation, n (%)	47 (69)	28 (67)	7 (78)	12 (71)	*1.000*
1st indication biopsy, time post-transplant, months*	3 (0-91)	3.5 (0-48)	3 (0-91)	2 (0-58)	*0.665*
Follow-up biopsy, time post-1st biopsy, months*	4 (0-25)	6 (0-24)	15 (0-25)	2 (0-18)	0.008*
Immunosuppression					
Tacrolismus, n (%)	60 (88)	38 (90)	9 (100)	13 (76)	*0.211*
Ciclosporine, n (%)	8 (12)	4 (10)	0 (0)	4 (24)	*0.211*
MMF/EC-MPA, n (%)	67 (99)	41 (98)	9 (100)	17 (100)	*1.000*
Donor characteristics					
Donor age, years*	56.5 (3-78)	54.5 (3-73)	54 (41-73)	59 (28-78)	*0.165*
Donor, male sex, n (%)	36 (53)	22 (52)	4 (44)	10 (59)	*0.776*
Immunocompatibility					
Total HLA mismatches*	7 (0-10)	6.5 (0-10)	6 (4-10)	8 (2-9)	*0.272*
Total PIRCHE-Score*	68.66 (0-195.43)	65.89 (0-195.43)	66.41 (33.47-130.44)	107.33 (36.68-175.62)	0.031*
PIRCHE-A	15.35 (0-69.37)	14.72 (0-69.37)	11 (3.74-30.92)	24.59 (0-51.71)	*0.110*
PIRCHE-B	13.93 (0-40.80)	12 (0-35.07)	12.52 (7.00-31.51)	21.23 (0-40.80)	*0.640*
PIRCHE-C	10.47 (0-50.00)	10.85 (0-31.44)	9.97 (1.00-23.94)	9 (0-50.00)	*0.598*
PIRCHE-DR	11.51 (0-43.47)	10.05 (0-37.11)	12 (5.00-31.38)	14.39 (2.39-43.47)	0.046*
PIRCHE-DQ	21.76 (0-84.91)	20.14 (0-84.91)	17.2 (0-36.64)	27 (6.00-60.13)	*0.052*
PIRCHE HLA-I	43.44 (0-133.86)	40.34 (0-133.86)	32.25 (19.00-84.17)	62.18 (0.04-93.05)	0.017*
PIRCHE HLA-II	32.98 (0-120.74)	29.39 (0-120.74)	31.99 (11.19-61.62)	39.9 (14.77-86.25)	0.017*

*median (range).

**Table 2:** Basic characteristics of 21 KTRs with TCMR in the first indication biopsy and grouping based on the results of the follow-up biopsy.

	Total	NR/BLR	ABMR	TCMR	*P*
	n=21	n=12	n=3	n=6	*NR/BLR vs. TCMR*
Recipient characteristics					
Recipient age, years*	51 (30-73)	46 (30-61)	55 (51-70)	56.5 (37-73)	*0.151*
Recipient, male sex, n (%)	15 (71)	9 (75)	3 (100)	3 (50)	*0.344*
Living donation, n (%)	4 (19)	2 (17)	0 (0)	2 (33)	*0.569*
Deceased donation, n (%)	17 (81)	10 (83)	3 (100)	4 (67)	*0.569*
1st indication biopsy, time post-transplant, months*	2 (0-46)	1.5 (0-28)	3 (2-46)	3.5 (0-8)	*1.000*
Follow-up biopsy, time post-1st biopsy, months*	1 (0-14)	1.5 (0-14)	1 (0-1)	0.5 (0-5)	*0.616*
Immunosuppression					
Tacrolismus, n (%)	19 (90)	10 (83)	3 (100)	6 (100)	*0.529*
Ciclosporine, n (%)	2 (10)	2 (17)	0 (0)	0 (0)	*0.529*
MMF/EC-MPA, n (%)	21 (100)	12 (100)	3 (100)	6 (100)	*1.000*
Donor characteristics					
Donor age, years*	55 (23-74)	57 (40-73)	70 (46-74)	50.5 (23-74)	*0.291*
Donor, male sex, n (%)	12 (57)	6 (50)	3 (100)	3 (50)	*1.000*
Immunocompatibility					
Total HLA mismatches*	7 (4-9)	6.5 (4-9)	6 (5-8)	7.5 (6-9)	*0.180*
Total PIRCHE-Score*	79.03 (16.00-233.55)	76.52 (32.30-233.55)	63.76 (16.00-134.97)	101.50 (36.88-126.44)	*0.385*
PIRCHE-A	13.03 (1.00-42.87)	14.62 (6.38-40.07)	4.28 (1.00-42.87)	11.75 (7.00-25.35)	*0.616*
PIRCHE-B	14 (0-53.40)	10.43 (0-53.40)	8 (7.00-21.03)	17.86 (12.00-24.69)	*0.335*
PIRCHE-C	12.64 (0-75.06)	10 (0-75.06)	19.48 (0-43.61)	19.00 (0.40-38.00)	*0.616*
PIRCHE-DR	15 (3.00-37.93)	13.5 (7.08-37.93)	14 (3.00-21.00)	16.76 (7.00-31.90)	*0.437*
PIRCHE-DQ	21.04 (4.00-47.39)	21.05 (9.73-47.39)	8.09 (4.00-19.00)	25.57 (8.91-37.86)	*1.000*
PIRCHE HLA-I	36 (7.09-148.23)	34.15 (7.09-148.23)	30.76 (9.00-107.51)	47.94 (20.98-73.00)	*0.553*
PIRCHE HLA-II	33 (7.00-85.32)	34.77 (17.43-85.32)	29.09 (7.00-33.00)	49.18 (15.91-59.9)	*0.750*

*median (range).

**Table 3:** Basic characteristics of 30 KTRs with ABMR in the first indication biopsy and grouping based on the results of the follow-up biopsy.

	Total	Deteriorating MVI "+"	Stable MVI "0"	Improving MVI "-"	*P + vs. -*
	n=30	n=7	n=12	n=11	
Recipient characteristics					
Recipient age, years*	48.5 (18-74)	43 (18-67)	41 (21-68)	57 (33-74)	*0.179*
Recipient, male sex, n (%)	21 (70)	5 (71)	10 (83)	6 (55)	*0.637*
Living donation, n (%)	14 (47)	3 (43)	5 (42)	6 (55)	*1.000*
Deceased donation, n (%)	16 (53)	4 (57)	7 (58)	5 (45)	*1.000*
1st indication biopsy, time post-transplant, months*	36.5 (0-110)	36 (0-108)	30 (0-110)	46 (0-75)	*1.000*
Follow-up biopsy, time post-1st biopsy, months*	2 (0-24)	6 (0-24)	0.5 (0-19)	1 (0-17)	*0.211*
Immunosuppression					
Tacrolismus, n (%)	21 (70)	3 (43)	10 (83)	8 (73)	*0.332*
Ciclosporine, n (%)	9 (30)	4 (57)	2 (17)	3 (27)	*0.332*
MMF/EC-MPA, n (%)	30 (100)	7 (100)	12 (100)	11 (100)	*1.000*
Donor characteristics					
Donor age, years*	53 (31-76)	50 (33-62)	53 (39-69)	56 (31-76)	*0.536*
Donor, male sex, n (%)	12 (40)	4 (57)	5 (42)	3 (27)	*0.332*
Immunocompatibility					
Total HLA mismatches*	7 (3-10)	6 (3-10)	6.5 (3-10)	8 (4-10)	*0.536*
Total PIRCHE-Score*	79.99 (25.37-200.32)	128.38 (47.25-192.60)	79.99 (25.37-180.27)	70.75 (30.00-200.32)	*0.151*
PIRCHE-A	18.13 (0-55.68)	24.22 (1.08-40.84)	15.72 (0-55.68)	20.25 (0-52.07)	*0.724*
PIRCHE-B	13.09 (0.28-42.77)	17.75 (2.11-34.59)	12.26 (0.28-22.98)	15.09 (5.67-42.77)	*0.425*
PIRCHE-C	12.72 (0-49.37)	19.7 (1.39-49.37)	13.14 (0-43.85)	11 (0-23.46)	*0.151*
PIRCHE-DR	15.01 (0-42.00)	21.05 (0-42.00)	17.05 (6.78-39.99)	11.26 (1.00-39.37)	*0.246*
PIRCHE-DQ	21.5 (0-65.58)	28.15 (10.98-65.58)	22.09 (0-61.04)	18.21 (0.02-43.41)	*0.151*
PIRCHE HLA-I	47.99 (4.58-118.3)	57 (4.58-107.96)	39.92 (11.62-91.28)	46.43 (14.04-118.3)	*0.179*
PIRCHE HLA-II	36.5 (6.00-101.03)	70 (14.00-86.63)	40.15 (13.76-101.03)	30.2 (6.00-82.78)	*0.086*

*median (range).

Group NR/BLR: 68 of 123 KTRs (55.3%) showed NR/BLR in the first indication biopsy. 42 of 68 KTRs (61.8%) also showed NR/BLR in the follow-up biopsy. 17 of 68 KTRs (25.0%) showed TCMR, and 9 of 68 KTRs (13.2%) showed ABMR in the follow-up biopsy. The median total PIRCHE-II score was 68.66 (range: 0.00-195.43) with PIRCHE-II-A of 15.35 (0.00-69.37), PIRCHE-II-B of 13.93 (0.00-40.80), PIRCHE-II-C of 10.47 (0.00-50.00), PIRCHE-II-DQ of 21.76 (0.00-84.91), and PIRCHE-II-DR of 11.51 (0.00-43.47).

Group TCMR: 21 of 123 KTRs (17.1%) showed TCMR in the first indication biopsy. In the follow-up biopsy, NR/BLR was found in 12 of 21 KTRs (57.1%), ongoing TCMR in 6 of 21 KTRs (28.6%), and ABMR in 3 of 21 KTRs (14.3%). The median total PIRCHE-II score was 79.03 (16.00-233.55) with PIRCHE-II-A of 13.03 (1.00-42.87), PIRCHE-II-B of 14.00 (0.00-53.40), PIRCHE-II-C of 12.64 (0.00-75.06), PIRCHE-II-DQ of 21.04 (4.00-47.39), and PIRCHE-II-DR of 15.00 (3.00-37.93).

Group ABMR: 34 of 123 KTRs (27.6%) showed ABMR in the first indication biopsy. In the follow-up biopsy, 3 of 34 KTRs (8.8%) showed NR/BLR, 1 of 34 KTRs (2.95%) showed TCMR, and 30 of 34 KTRs (88.2%) showed ongoing ABMR. Among these 30 KTRs with ongoing ABMR, 7 of 30 (23.3%) KTRs developed worsening, 12 of 30 KTRs (40.0%) developed no change, and 11 of 30 KTRs (36.7%) improved the MVI score (Banff classification: ptc + g). The median total PIRCHE-II score was 79.99 (25.37-200.32) with PIRCHE-II-A of 18.13 (0.00-55.68), PIRCHE-II-B of 13.09 (0.28-42.77), PIRCHE-II-C of 12.72 (0.00-49.36), PIRCHE-II-DQ of 21.50 (0.00-65.58), and PIRCHE-II-DR of 15.01 (0.00-42.00).

Overall, KTRs with TCMR or ABMR in the first indication biopsy showed higher total PIRCHE-II scores than KTRs with NR/BRL in the first indication biopsy (79.03 and 79.99 vs. 68.66).

### Impact of PIRCHE-II scores on histopathological findings in follow-up biopsies

**Figure 3:**
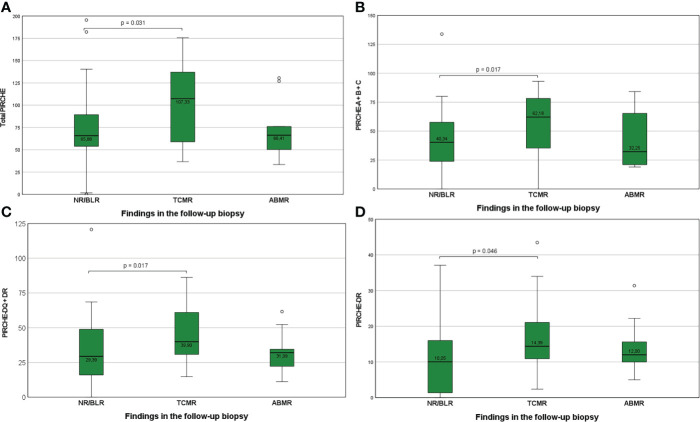
**(A-D)** Higher total PIRCHE-II scores **(A)**, PIRCHE-II scores for HLA-A, -B, and -C loci mismatches **(B)**, PIRCHE-II scores for HLA-DQ and -DR loci mismatches **(C)**, and PIRCHE-II scores for HLA-DR locus mismatches **(D)** among KTRs who develop TCMR compared to KTR with NR/BLR. Boxplots show median, interquartile range (IQR), and 95^th^ percentile.

Group NR/BLR: 17 of 68 KTRs (25.0%) who developed TCMR in the follow-up biopsy showed significantly higher median PIRCHE-II-A+B+C scores (p=0.017), PIRCHE-II-DQ+DR scores (p=0.017), PIRCHE-II-DR scores (p=0.046), and total PIRCHE-II scores (p=0.031), compared to 42 of 68 KTRs (61.8%) with NR/BLR in the follow-up biopsy (**Figures 3A–D**). No differences were observed between 9 of 68 KTRs (13.2%) who developed ABMR in the follow-up biopsy compared to KTRs with NR/BLR in the follow-up biopsy.

**Figure 4:**
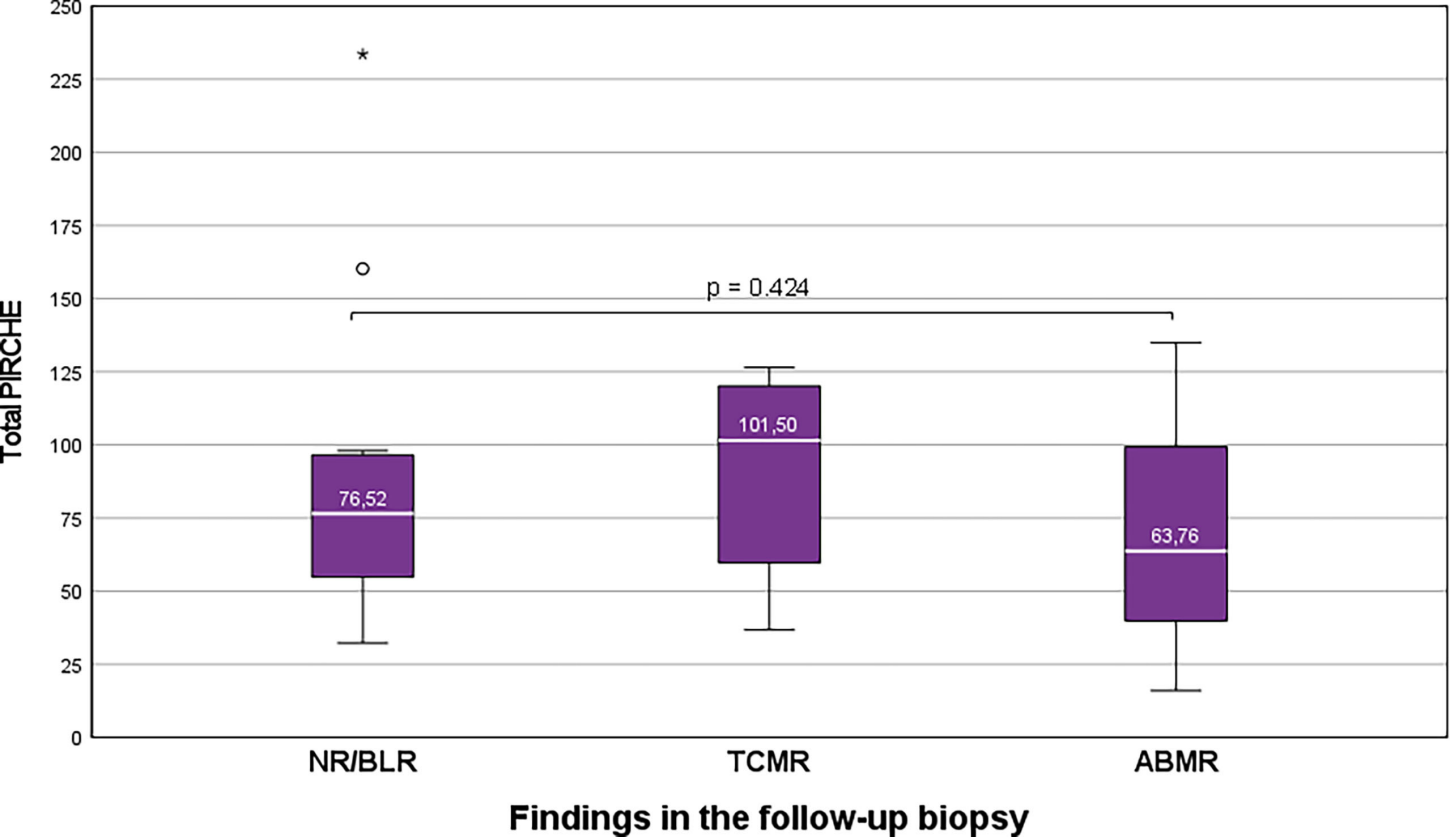
Higher total PIRCHE-II scores among KTRs who show ongoing TCMR than KTRs with NR/BLR/ABMR. Boxplots show median, interquartile range (IQR), and 95^th^ percentile The asterisk (*) marks the maximum outlier.

Group TCMR: 6 of 21 KTRs (28.6%), who showed ongoing TCMR in the follow-up biopsy, showed higher median total PIRCHE-II scores than the remaining 15 of 21 KTRs (71.4%) who showed NR/Borderline or ABMR in the follow-up biopsy (median 101.50 vs. 74.00; **Figure 4**).

**Figure 5:**
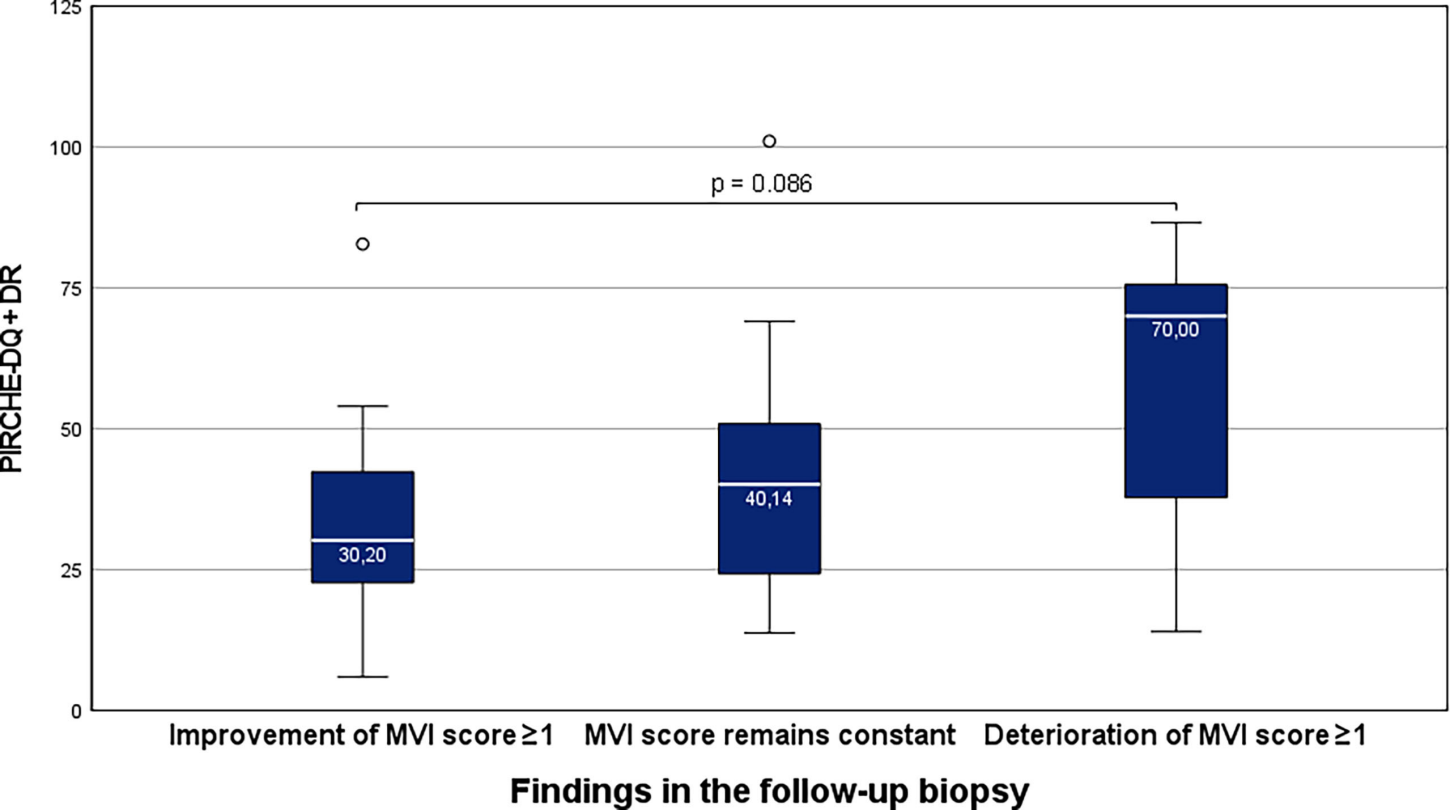
Higher total PIRCHE-II scores for the HLA-DQ and -DR loci mismatches among KTRs with ABMR, who show an increase in the MVI-score in the follow-up biopsy. Boxplots show median, interquartile range (IQR), and 95^th^ percentile.

Group ABMR: 7 of 30 KTRs (23.3%), who showed an increase in the MVI score in the follow-up biopsy, showed higher total PIRCHE-II scores compared to 23 of 30 KTRs (76.7), who showed stable or decreasing MVI scores in the follow-up biopsy (p=). This difference was more pronounced for the PIRCHE-II-DQ+DR (median 70.00 vs. 31.76; p=0.086) than for de PIRCHE-A+B+C (median 57.00 vs. 44.50; p=0.107; **Figure 5**).

## Discussion

In post-transplant care, the challenge is to find the optimal therapy between the development of immune-mediated injury and over-immunosuppression. The commonly accepted treatment strategies were highlighted in the recent comprehensive UNOS survey of kidney transplant programs in the United States (32). Recent research has undertaken significant efforts to find possible methods for risk stratification.

HLA epitope matching algorithms such as the PIRCHE-II scores presumably provide a more precise assessment of HLA compatibility between donor and recipient than antigen-based HLA-matching (33). The PIRCHE-II score is a marker of indirect T-cell alloreactivity, crucial for developing immune-mediated damage. The cohort analysis of more than 65,000 KTRs from the Collaborative Transplant Study suggested that PIRCHE-II scores might strongly predict 5-year death-censored kidney allograft loss (34). Our study evaluated whether the PIRCHE-II score is a predictive biomarker to forecast the histopathological changes of immune-mediated injury from a first indication biopsy to a follow-up biopsy and whether the PIRCHE-II score is appropriate for risk stratification.

Our results confirm the large discrepancy in immunological risk stratification using the number of HLA-antigen mismatches and the number of molecular HLA-epitope mismatches. Certain HLA-antigen mismatches can lead to severe clinical alloreactivity and are considered highly immunogenic HLA mismatches. Other HLA-antigen mismatches do not typically lead to clinical alloreactivity (11, 20–23). Therefore, determining the molecular HLA-epitope mismatch may be more accurate than just counting the number of HLA-antigen mismatches (11, 20), and the PIRCHE-II algorithm seems more suitable for stratifying the risk of immune-mediated injury. Geneugelijk et al. reported in 2018 that a higher PIRCHE-II score was strongly associated with a higher risk of kidney allograft failure. In addition, in their multivariate model, the predictive power of PIRCHE-II scores was stronger than that of HLA-antigen mismatches (17, 34). The predictive power of PIRCHE-II scores for the development of *de novo* DSA has been shown to be strongest for HLA-DQ and HLA-DR, followed by HLA-A and HLA-B (16).

KTRs with NR/BLR in a first indication biopsy were more likely to progress to TCMR/ABMR in the follow-up biopsy in case of higher PIRCHE-II scores. This result confirms our recently published hypothesis in a much larger cohort of 68 KTRs with follow-up biopsies (2). It strengthens our suggestion that PIRCHE-II scores have the potential as a prognostic biomarker for risk assessment in KTRs with BLR. In contrast to our previous work (2), we now find that high HLA class I and II-derived PIRCHE-II scores are similarly strongly associated with the occurrence of TCMR/ABMR during the course. This difference is probably because the current study has a significantly higher number of cases.

Furthermore, it seems immunologically more plausible that the risk for cellular allorecognition *via* the indirect pathway in the early phase after transplantation depends more on the HLA epitope mismatch load and the presence of highly immunogenic HLA-epitopes on the HLA antigen class itself. Setting a cutoff for the total PIRCHE-II score in the 83rd percentile identified 9 of 12 KTRs that developed TCMR in the follow-up biopsy. These KTRs may then be treated more aggressively, either with increased maintenance immunosuppression or steroid pulses in some instances, to prevent progression to TCMR/ABMR. Therefore, PIRCHE-II scores may help decide on an anti-rejection treatment at the time of an indication biopsy with BLR.

KTRs treated for TCMR, according to a first indication biopsy, were more likely to show ongoing TCMR in the follow-up biopsy in case of higher PIRCHE-II scores. This finding suggests that PIRCHE-II scores not only compare amino acid sequences but indeed discriminate the immunogenicity of certain HLA-epitopes. The approach to the initial anti-rejection therapy in KTRs with histologic evidence of TCMR is guided predominantly by the histopathologic severity of rejection. However, the supposed higher immunogenicity of certain HLA-epitopes may also be considered and explain why some KTRs show ongoing or treatment-resistant courses of TCMR despite similar treatment regimes. Since the number of steroid pulses, the use of T-cell depleting anti-rejection treatment, and the addition of a long, short, or no steroid tapering remains controversial and mostly rely on center-specific protocols, biomarkers to guide the intensity of anti-rejection treatment are of particular interest. There is no high-quality evidence to support one anti-rejection treatment approach over another. Therefore, PIRCHE-II scores may help decide on a more or less intense anti-rejection treatment at the time of an indication biopsy with TCMR, which should be addressed in prospective studies.

KTRs with ABMR in a first indication biopsy were more likely to show a progression of the MVI in the follow-up biopsy in case of higher HLA class II-derived PIRCHE-II scores. HLA class II-derived epitopes are of particular interest since the development of *de novo* DSA is predominantly directed against HLA class II (24). Higher PIRCHE-II scores indicate a greater risk for a higher number of highly immunogenic HLA epitope mismatches. DSA, directed against these highly immunogenic HLA epitope mismatches, may potentially result in more rapid progression and injury, as illustrated by the increased MVI scores in our study. Therefore, our data emphasize the importance of HLA class II matching to prevent the development of *de novo* DSA, but in the case of ABMR, also progression. Importantly, our clinical observations thus indicate for the first time on a histopathological level that PIRCHE-II scores not only compare amino acid sequences but indeed discriminate the immunogenicity of certain HLA-epitopes. This observation should be applied retrospectively and prospectively to investigate whether PIRCHE-II scores can predict treatment response in ABMR. This seems particularly interesting for the ongoing studies on the use of anti-IL-6 treatment in chronic active ABMR. Therefore, PIRCHE-II scores should be considered a biomarker to evaluate the impact of different ABMR treatments.

The cause of the progression of immune-related injury to the kidney allograft is assumed to be multifactorial. Thus, it is even more important and surprising that PIRCHE-II scores impacted histopathological changes from a first indication biopsy to a second follow-up biopsy for all rejection types. This suggests that the impact of histocompatibility on severity, progression, and response to treatment of BLR, TCMR, and ABMR has been underestimated over the past. Our study is another important contribution to the field of computational prediction of biomarkers in the pathogenesis of kidney allograft rejection. The PIRCHE-II score will likely be important in the assessment of immunological pathways as well as in the evaluation of the therapeutic response to new target drugs (35).

Our study has some limitations. Firstly, the sample size of all three groups is comparatively small. Therefore, the hypothesis needs to be validated in a larger cohort. Secondly, the retrospective single-center design might limit the generalizability of our results. However, our study also has several strengths. Our KTRs have been excellently characterized over many years. A standardized protocol of immunosuppression is established. A close functional and clinical monitoring post-transplantation facilitated us to gain a very high data density. All kidney allograft biopsies were also examined and evaluated by the same nephropathologist, preventing interpersonal variability in histopathological examination.

In summary, this is the first single-center analysis of 123 KTRs and the impact of PIRCHE-II scores on the histopathological course from a first indication to a second follow-up biopsy. Our data suggest that PIRCHE-II scores are an independent predictor of histopathological progression. The use of biomarkers to identify KTRs at an increased risk for allorecognition is necessary for individualized treatment strategies. Our findings strengthen the potential of PIRCHE-II scores as a biomarker to predict the risk of immune-mediated injury from a first indication biopsy to a follow-up biopsy. Interestingly, this predictive value of the PIRCHE-II score is shown for all histological rejection types of NR/BLR, TCMR, and ABMR. Future randomized controlled trials using the PIRCHE-II score as a biomarker, possibly in combination with other promising biomarkers such as quantification of preformed alloreactive T cells or donor-derived cell-free DNA, could allow for individualized treatment strategies for KTRs with histologically confirmed rejection. Importantly, considering the apparent limitations of our single-center analysis, cautious interpretation and further validation are warranted.

## Data availability statement

The raw data supporting the conclusions of this article will be made available by the authors, without undue reservation.

## Ethics statement

The studies involving human participants were reviewed and approved by Cantonal Ethics Commitee Zurich. The patients/participants provided their written informed consent to participate in this study.

## Author contributions

TSp: Participated in data collection, writing of the paper, the performance of the research, and data analysis. LM: Participated in data collection. YK: Participated in data collection. JN: Participated in data collection and writing of the paper. SM: Participated in the writing of the paper. TSc: Participated in research design, writing of the paper, the performance of the research, data analysis, and data collection. All authors contributed to the article and approved the submitted version.

## Conflict of interest

The authors declare that the research was conducted in the absence of any commercial or financial relationships that could be construed as a potential conflict of interest.

## Publisher’s note

All claims expressed in this article are solely those of the authors and do not necessarily represent those of their affiliated organizations, or those of the publisher, the editors and the reviewers. Any product that may be evaluated in this article, or claim that may be made by its manufacturer, is not guaranteed or endorsed by the publisher.

## Abbreviations

ABMR, antibody-mediated rejection; APC, antigen-presenting cell; Banff-terms: I, interstitial inflammation; TI, total inflammation; T, tubulitis; V, intimal arteritis; G, glomerulitis; PTC, peritubular capillaritis; C4D, c4d deposition; CI, interstitial fibrosis; CT tubular, atrophy; CV, vascular fibrous intimal thickening; CG, transplant glomerulopathy; AH, arteriolar hyalinosis; AAH, hyaline arteriolar thickening; MM, mesangial matrix increase; BKV, polyomavirus BK; BLR, borderline rejection; CMV, cytomegalovirus; CNI, calcineurin inhibitor; DGF, delayed graft function; DSA, donor-specific anti HLA-antibodies; EGFR, estimated glomerular filtration rate; HLA, human leukocyte antigen; IL-2, interleukin-2; KTRs, kidney transplant recipients; MMF, mycophenolate mofetil; MPA, mycophenolic acid; MVI, microvascular inflammation; NR, no rejection; PEX, plasma exchange; PIRCHE-II, Predicted Indirectly ReCognizable HLA Epitopes; PKD, Polycystic kidney disease; TCMR, T-cell mediated rejection.
