# Supplementary material for: PIRCHE-II scores prove useful as a predictive biomarker among kidney transplant recipients with rejection: An analysis of indication and follow-up biopsies
Source: Front Immunol. 2022 Aug 17;13:949933. doi: 10.3389/fimmu.2022.949933 (PMC9428698; doi:10.3389/fimmu.2022.949933)
Supplement: Supplementary file 1 [file DataSheet_1.docx]

**Supplement Table 1A-C.** Basic characteristics (A), characteristics at 1^st^ biopsy (B) and at 2^nd^ biopsy (C) of 123 KTRs with a first indication biopsy.

**A**

|  | **Total**  n=123 | **NR/BLR**  n=68 | **ABMR**  n=34 | **TCMR**  n=21 | ***P***  *all groups* | ***P***  *NR/BLR vs. ABMR* | ***P***  *NR/BLR vs. TCMR* | ***P***  *ABMR vs. TCMR* |
| --- | --- | --- | --- | --- | --- | --- | --- | --- |
| **Basic characteristics** |  |  |  |  |  |  |  |  |
| **Recipient characteristics** |  |  |  |  |  |  |  |  |
| Recipient age, years* | 52 (18-75) | 54.5 (18-75) | 48.5 (18-74) | 51 (30-73) | *0.286* | *0.131* | *0.448* | *0.499* |
| Recipient, male sex, n (%) | 74 (60) | 36 (53) | 23 (68) | 15 (71) | *0.205* | *0.203* | *0.207* | *1.000* |
| Renal disease, n (%) |  |  |  |  |  |  |  |  |
| ·       Diabetic | 12 (10) | 10 (15) | 2 (6) | 0 (0) | *0.104* | *0.328* | *0.109* | *0.519* |
| ·       Hypertensive | 8 (7) | 4 (6) | 2 (6) | 2 (10) | *0.781* | *1.000* | *0.623* | *0.632* |
| ·       PKD | 15 (12) | 7 (10) | 6 (18) | 2 (10) | *0.564* | *0.350* | *1.000* | *0.696* |
| ·       Glomerular disease | 38 (31) | 25 (37) | 9 (26) | 4 (19) | *0.263* | *0.375* | *0.184* | *0.745* |
| ·       Others | 30 (24) | 12 (18) | 9 (26) | 9 (43) | *0.064* | *0.311* | *0.036** | *0.246* |
| ·       Unknown | 20 (16) | 10 (15) | 6 (18) | 4 (19) | *0.842* | *0.775* | *0.733* | *1.000* |
| Living donation, n (%) | 41 (33) | 21 (31) | 16 (47) | 4 (19) | *0.087* | *0.129* | *0.407* | *0.046** |
| Deceased donation, n (%) | 82 (67) | 47 (69) | 18 (53) | 17 (81) | *0.087* | *0.129* | *0.407* | *0.046** |
| AB0-incompatible, n (%) | 10 (8) | 7 (10) | 0 (0) | 3 (14) | *0.062* | *0.092* | *0.695* | *0.051* |
| Kidney/Pancreas-transplantation, n (%) | 2 (2) | 1 (1) | 1 (3) | 0 (0) | *1.000* | *1.000* | *1.000* | *1.000* |
| Retransplantation, n (%) | 17 (14) | 9 (13) | 6 (18) | 2 (10) | *0.771* | *0.564* | *1.000* | *0.696* |
| Cold ischemia time* | 572 (195-1'618) | 592 (267-1'618) | 555 (276-847) | 535 (195-1'000) | *0.581* | *0.907* | *0.784* | *0.546* |
| Tumor before transplantation, n (%) | 10 (8) | 8 (12) | 1 (3) | 1 (5) | *0.359* | *0.266* | *0.680* | *1.000* |
| Tumor after transplantation, n (%) | 3 (2) | 2 (3) | 1 (3) | 0 (0) | *1.000* | *1.000* | *1.000* | *1.000* |
| **Immunosuppression** |  |  |  |  |  |  |  |  |
| ·       Tacrolismus, n (%) | 103 (84) | 60 (88) | 24 (71) | 19 (90) | *0.072* | *0.051* | *1.000* | *0.104* |
| ·       Ciclosporine, n (%) | 20 (16) | 8 (12) | 10 (29) | 2 (10) | *0.072* | *0.051* | *1.000* | *0.104* |
| ·       Cellcept (MMF), n (%) | 102 (83) | 61 (90) | 26 (76) | 15 (71) | *0.063* | *0.135* | *0.070* | *0.755* |
| ·       Myofortic (MPA), n (%) | 20 (16) | 6 (9) | 8 (24) | 6 (29) | *0.034** | *0.065* | *0.031** | *0.755* |
| **Donor characteristics** |  |  |  |  |  |  |  |  |
| Donor age, years* | 56 (3-78) | 56.5 (3-78) | 56 (31-76) | 55 (23-74) | *0.834* | *0.804* | *0.602* | *0.609* |
| Donor, male sex, n (%) | 62 (50) | 36 (53) | 14 (41) | 12 (57) | *0.402* | *0.298* | *0.806* | *0.279* |
| **Immunocompatibility** |  |  |  |  |  |  |  |  |
| Total HLA mismatches* | 7 (0-10) | 7 (0-10) | 7 (3-10) | 7 (4-9) | *0.988* | *0.954* | *0.965* | *0.806* |
| Total PIRCHE-Score* | 71.96 (0-233.55) | 68.66 (0-195.43) | 78.78 (25.37-200.32) | 79.03 (16.00-233.55) | *0.617* | *0.327* | *0.667* | *0.782* |
| ·       PIRCHE-A | 14.89 (0-69.37) | 15.35 (0-69.37) | 15.72 (0-55.68) | 13.03 (1.00-42.87) | *0.865* | *0.929* | *0.595* | *0.678* |
| ·       PIRCHE-B | 13.85 (0-53.40) | 13.93 (0-40.80) | 13.2 (0.28-42.77) | 14 (0-53.40) | *0.974* | *0.986* | *0.816* | *0.862* |
| ·       PIRCHE-C | 11.1 (0-75.06) | 10.47 (0-50.00) | 14.05 (0-49.37) | 12.64 (0-75.06) | *0.449* | *0.205* | *0.543* | *0.862* |
| ·       PIRCHE-DR | 13.81 (0-43.47) | 11.51 (0-43.47) | 14.69 (0-42.00) | 15 (3.00-37.93) | *0.096* | *0.070* | *0.099* | *0.938* |
| ·       PIRCHE-DQ | 21.06 (0-84.91) | 21.76 (0-84.91) | 20.83 (0-65.58) | 21.04 (4.00-47.39) | *0.938* | *0.755* | *0.798* | *0.993* |
| ·       PIRCHE HLA-I | 43.44 (0-148.23) | 43.44 (0-133.86) | 46.7 (4.58-118.30) | 36 (7.09-148.23) | *0.865* | *0.683* | *0.862* | *0.591* |
| ·       PIRCHE HLA-II | 32 (0-120.74) | 32.98 (0-120.74) | 33.24 (6.00-101.03) | 33 (7.00-85.32) | *0.477* | *0.268* | *0.423* | *1.000* |
| Preformed DSA, n (%) | 50 (41) | 26 (38) | 18 (53) | 6 (29) | *0.175* | *0.204* | *0.604* | *0.098* |

*median (range)

**B**

|  | **Total**  n=123 | **NR/BLR**  n=68 | **ABMR**  n=34 | **TCMR**  n=21 | ***P***  *all groups* | ***P***  *NR/BLR vs. ABMR* | ***P***  *NR/BLR vs. TCMR* | ***P***  *ABMR vs. TCMR* |
| --- | --- | --- | --- | --- | --- | --- | --- | --- |
| **1. Renal biopsy** |  |  |  |  |  |  |  |  |
| Time post-transplant, months* | 5 (0-110) | 3 (0-91) | 36.5 (0-110) | 2 (0-46) | *0.000** | *0.000** | *0.608* | *0.001** |
| De-novo DSA, n (%) | 61 (50) | 28 (41) | 25 (74) | 8 (38) | *0.004** | *0.003** | *1.000* | *0.012** |
| **Kidney transplant function** |  |  |  |  |  |  |  |  |
| Creatinine baseline 1 year before biopsy, μmol/L* | 167.8 (52.00-514.67) | 167.56 (52.00-514.67) | 160.38 (71.00-406.33) | 180.62 (58.33-452.67) | *0.867* | *0.901* | *0.588* | *0.697* |
| Creatinine at biopsy, μmol/L* | 244.03 (84.00-865.00) | 247.26 (84.00-636.00) | 221.55 (93.00-865.00) | 267.83 (89.00-527.00) | *0.162* | *0.310* | *0.286* | *0.034** |
| Proteinuria baseline 1 year before biopsy, mg/day* | 466.97 (0-11'930.00) | 603.64 (0-11'930.00) | 302.38 (0-1'450.00) | 295.88 (30.00-1'113.33) | *0.814* | *0.721* | *0.524* | *0.851* |
| Proteinuria at biopsy, mg/day* | 1'871.21 (0-55'130.00) | 2'190.17 (0-55'130.00) | 2'069.64 (60.00-4'1930.00) | 571.58 (70.00-3'310.00) | *0.494* | *0.496* | *0.266* | *0.618* |
| Creatinine baseline 1 year after transplantation, μmol/L* | 115.33 (52.00-271.00) | 112.38 (52.00-271.00) | 110.24 (53.00-202.33) | 132.59 (53.67-263.67) | *0.120* | *0.864* | *0.067* | *0.046** |
| Proteinuria baseline 1 year after transplantation, mg/day* | 161.69 (0-1'693.33) | 196.87 (0-1'693.33) | 88.24 (0-303.33) | 168.41 (0-673.33) | *0.073* | *0.026** | *0.871* | *0.120* |
| Creatinine: Worsening between first and third baseline, n (%) | 45 (37) | 27 (40) | 9 (26) | 9 (43) | *0.361* | *0.272* | *0.805* | *0.246* |
| Proteinuria: Worsening between first and third baseline, n (%) | 39 (32) | 20 (29) | 12 (35) | 7 (33) | *0.834* | *0.652* | *0.789* | *1.000* |
| Creatinine: Stable between first and third baseline, n (%) | 50 (41) | 25 (37) | 17 (50) | 8 (38) | *0.416* | *0.210* | *1.000* | *0.419* |
| Proteinuria Stable between first and third baseline, n (%) | 20 (16) | 12 (18) | 6 (18) | 2 (10) | *0.754* | *1.000* | *0.505* | *0.696* |
| Creatinine: Improving between first and third baseline, n (%) | 22 (18) | 13 (19) | 5 (15) | 4 (19) | *0.900* | *0.784* | *1.000* | *0.719* |
| Proteinuria: Improving between first and third baseline, n (%) | 33 (27) | 18 (26) | 7 (21) | 8 (38) | *0.385* | *0.628* | *0.410* | *0.215* |
| DGF, n (%) | 25 (20) | 15 (22) | 5 (15) | 5 (24) | *0.648* | *0.439* | *1.000* | *0.480* |
| Dialysis treatment after transplantation, n (%) | 32 (26) | 17 (25) | 9 (26) | 6 (29) | *0.960* | *1.000* | *0.779* | *1.000* |

*median (range)

**C**

|  | **Total**  n=123 | **NR/BLR**  n=68 | **ABMR**  n=34 | **TCMR**  n=21 | ***P***  *all groups* | ***P***  *NR/BLR vs. ABMR* | ***P***  *NR/BLR vs. TCMR* | ***P***  *ABMR vs. TCMR* |
| --- | --- | --- | --- | --- | --- | --- | --- | --- |
| **2. Renal biopsy** |  |  |  |  |  |  |  |  |
| Time post-1st biopsy, months* | 3 (0-25) | 4 (0-25) | 2 (0-24) | 1 (0-14) | *0.017** | *0.123* | *0.006** | *0.264* |
| **Kidney transplant function** |  |  |  |  |  |  |  |  |
| Creatinine baseline 1 year before second biopsy, μmol/L* | 175.98 (65.67-549.00) | 176.21 (65.67-549.00) | 167.4 (96.67-370.00) | 187.08 (82.33-435.00) | *0.650* | *0.826* | *0.416* | *0.387* |
| Creatinine at second biopsy, μmol/L* | 239.41 (72.00-1'090.00) | 261.76 (72.00-1'090.00) | 189.42 (97.00-368.00) | 249.55 (106.00-596.00) | *0.107* | *0.065* | *0.716* | *0.065* |
| Creatinine baseline 1 year after second biopsy, μmol/L* | 171.94 (54.33-497.00) | 172.38 (61.33-476.33) | 159.3 (61.00-497.00) | 189.25 (54.33-438.67) | *0.481* | *0.374* | *0.637* | *0.229* |
| Proteinuria baseline 1 year before second biopsy, mg/day* | 702.59 (0-11'563.33) | 881.75 (0-11'563.33) | 496.42 (0-2'240.00) | 414.63 (0-2'066.67) | *0.868* | *0.604* | *0.854* | *0.772* |
| Proteinuria at second biopsy, mg/day* | 1'367.77 (0-37'960.00) | 1'255.41 (0-14'890.00) | 2'125.45 (0-37'960.00) | 359.44 (90.00-1'260.00) | *0.681* | *0.874* | *0.400* | *0.460* |
| Proteinuria baseline 1 year after second biopsy, mg/day* | 553.63 (0-5'290.00) | 530.35 (0-4'545.00) | 715.75 (0-5'290.00) | 383.02 (0-3'100.00) | *0.578* | *0.476* | *0.536* | *0.346* |

*median (range)

**Supplement Table 2A-C.** Basic characteristics (A), characteristics at 1^st^ biopsy (B) and at 2^nd^ biopsy (C) of 68 KTRs with NR/BLR in the first indication biopsy.

**A**

|  | **Total**  n=68 | **NR/BLR**  n=42 | **ABMR**  n=9 | **TCMR**  n=17 | **P**  all groups | **P**  NR/BLR vs. ABMR | **P**  NR/BLR vs. TCMR | **P**  ABMR vs. TCMR |
| --- | --- | --- | --- | --- | --- | --- | --- | --- |
| **Recipient characteristics** |  |  |  |  |  |  |  |  |
| Recipient age, years* | 54.5 (18-75) | 54.5 (18-75) | 52 (30-66) | 58 (26-69) | 0.464 | 0.855 | 0.315 | 0.200 |
| Recipient, male sex, n (%) | 36 (53) | 19 (45) | 7 (78) | 10 (59) | 0.188 | 0.140 | 0.399 | 0.418 |
| Renal disease, n (%) |  |  |  |  |  |  |  |  |
| ·       Diabetic | 10 (15) | 7 (17) | 0 (0) | 3 (18) | 0.616 | 0.328 | 1.000 | 0.529 |
| ·       Hypertensive | 4 (6) | 3 (7) | 0 (0) | 1 (6) | 1.000 | 1.000 | 1.000 | 1.000 |
| ·       PKD | 7 (10) | 6 (14) | 1 (11) | 0 (0) | 0.233 | 1.000 | 0.168 | 0.346 |
| ·       Glomerular disease | 25 (37) | 15 (36) | 5 (56) | 5 (29) | 0.417 | 0.289 | 0.766 | 0.234 |
| ·       Others | 12 (18) | 4 (10) | 3 (33) | 5 (29) | 0.065 | 0.095 | 0.103 | 1.000 |
| ·       Unknown | 10 (15) | 7 (17) | 0 (0) | 3 (18) | 0.616 | 0.328 | 1.000 | 0.529 |
| Living donation, n (%) | 21 (31) | 14 (33) | 2 (22) | 5 (29) | 0.929 | 0.701 | 1.000 | 1.000 |
| Deceased donation, n (%) | 47 (69) | 28 (67) | 7 (78) | 12 (71) | 0.929 | 0.701 | 1.000 | 1.000 |
| AB0-incompatible, n (%) | 7 (10) | 5 (12) | 1 (11) | 1 (6) | 0.859 | 1.000 | 0.662 | 1.000 |
| Kidney/Pancreas-transplantation, n (%) | 1 (1) | 0 (0) | 0 (0) | 1 (6) | 0.382 | 1.000 | 0.288 | 1.000 |
| Retransplantation, n (%) | 9 (13) | 6 (14) | 2 (22) | 1 (6) | 0.449 | 0.619 | 0.661 | 0.268 |
| Cold ischemia time* | 592 (267-1'618) | 594 (267-1'618) | 526 (415-659) | 625.17 (312-1'195) | 0.716 | 0.586 | 0.652 | 0.482 |
| Tumor before transplantation, n (%) | 8 (12) | 6 (14) | 1 (11) | 1 (6) | 0.859 | 1.000 | 0.661 | 1.000 |
| Tumor after transplantation, n (%) | 2 (3) | 0 (0) | 1 (11) | 1 (6) | 0.143 | 0.176 | 0.288 | 1.000 |
| **Immunosuppression** |  |  |  |  |  |  |  |  |
| ·       Tacrolismus, n (%) | 60 (88) | 38 (90) | 9 (100) | 13 (76) | 0.239 | 1.000 | 0.211 | 0.263 |
| ·       Ciclosporine, n (%) | 8 (12) | 4 (10) | 0 (0) | 4 (24) | 0.239 | 1.000 | 0.211 | 0.263 |
| ·       Cellcept (MMF), n (%) | 61 (90) | 38 (90) | 8 (89) | 15 (88) | 1.000 | 1.000 | 1.000 | 1.000 |
| ·       Myofortic (MPA), n (%) | 6 (9) | 3 (7) | 1 (11) | 2 (12) | 0.572 | 0.552 | 0.620 | 1.000 |
| **Donor characteristics** |  |  |  |  |  |  |  |  |
| Donor age, years* | 56.5 (3-78) | 54.5 (3-73) | 54 (41-73) | 59 (28-78) | 0.374 | 0.706 | 0.165 | 0.525 |
| Donor, male sex, n (%) | 36 (53) | 22 (52) | 4 (44) | 10 (59) | 0.778 | 0.726 | 0.776 | 0.683 |
| **Immunocompatibility** |  |  |  |  |  |  |  |  |
| Total HLA mismatches* | 7 (0-10) | 6.5 (0-10) | 6 (4-10) | 8 (2-9) | 0.490 | 0.894 | 0.272 | 0.339 |
| Total PIRCHE-Score* | 68.66 (0-195.43) | 65.89 (0-195.43) | 66.41 (33.47-130.44) | 107.33 (36.68-175.62) | 0.076 | 0.818 | 0.031* | 0.107 |
| ·       PIRCHE-A | 15.35 (0-69.37) | 14.72 (0-69.37) | 11 (3.74-30.92) | 24.59 (0-51.71) | 0.182 | 0.688 | 0.110 | 0.107 |
| ·       PIRCHE-B | 13.93 (0-40.80) | 12 (0-35.07) | 12.52 (7.00-31.51) | 21.23 (0-40.80) | 0.154 | 0.488 | 0.640 | 0.312 |
| ·       PIRCHE-C | 10.47 (0-50.00) | 10.85 (0-31.44) | 9.97 (1.00-23.94) | 9 (0-50.00) | 0.858 | 1.000 | 0.598 | 0.711 |
| ·       PIRCHE-DR | 11.51 (0-43.47) | 10.05 (0-37.11) | 12 (5.00-31.38) | 14.39 (2.39-43.47) | 0.107 | 0.277 | 0.046* | 0.634 |
| ·       PIRCHE-DQ | 21.76 (0-84.91) | 20.14 (0-84.91) | 17.2 (0-36.64) | 27 (6.00-60.13) | 0.116 | 0.836 | 0.052 | 0.120 |
| ·       PIRCHE HLA-I | 43.44 (0-133.86) | 40.34 (0-133.86) | 32.25 (19.00-84.17) | 62.18 (0.04-93.05) | 0.051 | 1.000 | 0.017* | 0.120 |
| ·       PIRCHE HLA-II | 32.98 (0-120.74) | 29.39 (0-120.74) | 31.99 (11.19-61.62) | 39.9 (14.77-86.25) | 0.046* | 0.725 | 0.017* | 0.085 |
| Preformed DSA, n (%) | 26 (38) | 17 (40) | 5 (56) | 4 (24) | 0.240 | 0.474 | 0.249 | 0.194 |

*median (range)

**B**

|  | **Total**  n=68 | **NR/BLR**  n=42 | **ABMR**  n=9 | **TCMR**  n=17 | **P**  all groups | **P**  NR/BLR vs. ABMR | **P**  NR/BLR vs. TCMR | **P**  ABMR vs. TCMR |
| --- | --- | --- | --- | --- | --- | --- | --- | --- |
| **1. Renal biopsy** |  |  |  |  |  |  |  |  |
| Time post-transplant, months* | 3 (0-91) | 3.5 (0-48) | 3 (0-91) | 2 (0-58) | 0.400 | 0.266 | 0.665 | 0.200 |
| ·       <6 months, n (%) | 46 (68) | 27 (64) | 5 (56) | 14 (82) | 0.254 | 0.711 | 0.222 | 0.188 |
| ·       6-12 months, n (%) | 9 (13) | 8 (19) | 1 (11) | 0 (0) | 0.147 | 1.000 | 0.090 | 0.346 |
| ·       13-60 months, n (%) | 11 (16) | 7 (17) | 1 (11) | 3 (18) | 1.000 | 1.000 | 1.000 | 1.000 |
| ·       >60 months, n (%) | 2 (3) | 0 (0) | 2 (22) | 0 (0) | 0.016* | 0.028* | 1.000 | 0.111 |
| Indication for 1. biopsy |  |  |  |  |  |  |  |  |
| ·       eGFR, n (%) | 31 (46) | 18 (43) | 4 (44) | 9 (53) | 0.830 | 1.000 | 0.569 | 1.000 |
| ·       Proteinuria, n (%) | 17 (25) | 11 (26) | 3 (33) | 3 (18) | 0.720 | 0.692 | 0.737 | 0.628 |
| ·       eGFR and proteinuria, n (%) | 19 (28) | 13 (31) | 2 (22) | 4 (24) | 0.794 | 0.709 | 0.753 | 1.000 |
| ·       DSA, n (%) | 0 (0) | 0 (0) | 0 (0) | 0 (0) | 1.000 | 1.000 | 1.000 | 1.000 |
| ·       eGFR and BKV, n (%) | 0 (0) | 0 (0) | 0 (0) | 0 (0) | 1.000 | 1.000 | 1.000 | 1.000 |
| ·       Other, n (%) | 1 (1) | 0 (0) | 0 (0) | 1 (6) | 0.382 | 1.000 | 0.288 | 1.000 |
| Banff-Score, n (%) |  |  |  |  |  |  |  |  |
| ·       t-lesion ≥ 2 | 7 (10) | 4 (10) | 1 (11) | 2 (12) | 1.000 | 1.000 | 1.000 | 1.000 |
| ·       t-lesion = 1 | 21 (31) | 12 (29) | 1 (11) | 8 (47) | 0.173 | 0.417 | 0.228 | 0.098 |
| ·       i-lesion ≥ 2 | 2 (3) | 1 (2) | 1 (11) | 0 (0) | 0.309 | 0.325 | 1.000 | 0.346 |
| ·       i-lesion = 1 | 6 (9) | 3 (7) | 0 (0) | 3 (18) | 0.444 | 1.000 | 0.341 | 0.529 |
| ·       ti-lesion ≥ 2 | 6 (9) | 2 (5) | 4 (44) | 0 (0) | 0.004* | 0.006* | 1.000 | 0.008* |
| ·       ti-lesion = 1 | 11 (16) | 7 (17) | 0 (0) | 4 (24) | 0.363 | 0.328 | 0.713 | 0.263 |
| ·       ptc-lesion ≥ 2 | 2 (3) | 1 (2) | 1 (11) | 0 (0) | 0.309 | 0.325 | 1.000 | 0.346 |
| ·       ptc-lesion = 1 | 2 (3) | 1 (2) | 0 (0) | 1 (6) | 0.622 | 1.000 | 0.497 | 1.000 |
| ·       v-lesion ≥ 2 | 0 (0) | 0 (0) | 0 (0) | 0 (0) | 1.000 | 1.000 | 1.000 | 1.000 |
| ·       v-lesion = 1 | 0 (0) | 0 (0) | 0 (0) | 0 (0) | 1.000 | 1.000 | 1.000 | 1.000 |
| ·       cv-lesion ≥ 2 | 1 (1) | 0 (0) | 1 (11) | 0 (0) | 0.132 | 0.176 | 1.000 | 0.346 |
| ·       cv-lesion = 1 | 6 (9) | 2 (5) | 0 (0) | 4 (24) | 0.067 | 1.000 | 0.052 | 0.263 |
| ·       g-lesion ≥ 2 | 2 (3) | 1 (2) | 0 (0) | 1 (6) | 0.622 | 1.000 | 0.497 | 1.000 |
| ·       g-lesion = 1 | 1 (1) | 1 (2) | 0 (0) | 0 (0) | 1.000 | 1.000 | 1.000 | 1.000 |
| ·       cg-lesion ≥ 2 | 3 (4) | 2 (5) | 0 (0) | 1 (6) | 1.000 | 1.000 | 1.000 | 1.000 |
| ·       cg-lesion = 1 | 1 (1) | 0 (0) | 1 (11) | 0 (0) | 0.132 | 0.176 | 1.000 | 0.346 |
| ·       mm-lesion ≥ 2 | 3 (4) | 2 (5) | 0 (0) | 1 (6) | 1.000 | 1.000 | 1.000 | 1.000 |
| ·       mm-lesion = 1 | 2 (3) | 0 (0) | 1 (11) | 1 (6) | 0.143 | 0.176 | 0.288 | 1.000 |
| ·       ci-lesion ≥ 2 | 7 (10) | 2 (5) | 3 (33) | 2 (12) | 0.039* | 0.033* | 0.571 | 0.302 |
| ·       ci-lesion = 1 | 22 (32) | 15 (36) | 2 (22) | 5 (29) | 0.807 | 0.699 | 0.766 | 1.000 |
| ·       ct-lesion ≥ 2 | 7 (10) | 2 (5) | 3 (33) | 2 (12) | 0.039* | 0.033* | 0.571 | 0.302 |
| ·       ct-lesion = 1 | 44 (65) | 30 (71) | 3 (33) | 11 (65) | 0.103 | 0.052 | 0.756 | 0.218 |
| ·       ah-lesion ≥ 2 | 16 (24) | 9 (21) | 4 (44) | 3 (18) | 0.312 | 0.208 | 1.000 | 0.188 |
| ·       ah-lesion = 1 | 33 (49) | 18 (43) | 3 (33) | 12 (71) | 0.095 | 0.720 | 0.084 | 0.103 |
| ·       aah-lesion ≥ 2 | 8 (12) | 5 (12) | 2 (22) | 1 (6) | 0.409 | 0.592 | 0.662 | 0.268 |
| ·       aah-lesion = 1 | 3 (4) | 2 (5) | 0 (0) | 1 (6) | 1.000 | 1.000 | 1.000 | 1.000 |
| ·       cd4-lesion ≥ 2 | 4 (6) | 3 (7) | 0 (0) | 1 (6) | 1.000 | 1.000 | 1.000 | 1.000 |
| ·       cd4-lesion = 1 | 1 (1) | 1 (2) | 0 (0) | 0 (0) | 1.000 | 1.000 | 1.000 | 1.000 |
| **Induction Immunsuppression** |  |  |  |  |  |  |  |  |
| Steroid, n (%) | 0 (0) | 0 (0) | 0 (0) | 0 (0) | 1.000 | 1.000 | 1.000 | 1.000 |
| Other, n (%) | 0 (0) | 0 (0) | 0 (0) | 0 (0) | 1.000 | 1.000 | 1.000 | 1.000 |
| **Kidney transplant function** |  |  |  |  |  |  |  |  |
| Creatinine baseline 1 year before biopsy, μmol/L* | 167.56 (52.00-514.67) | 165.71 (64.33-407.00) | 200.11 (81.67-514.67) | 154.9 (52.00-370.33) | 0.350 | 0.173 | 0.933 | 0.200 |
| Creatinine at biopsy, μmol/L* | 247.26 (84.00-636.00) | 234.82 (84.00-636.00) | 244.22 (164.00-475.00) | 279.6 (92.00-613.00) | 0.477 | 0.373 | 0.323 | 0.958 |
| Proteinuria baseline 1 year before biopsy, mg/day* | 603.64 (0-11'930.00) | 621.67 (0-11'930.00) | 822.41 (113.33-3'085.00) | 406.41 (30.00-1'756.67) | 0.593 | 0.364 | 0.643 | 0.471 |
| Proteinuria at biopsy, mg/day* | 2190.17 (0-55'130.00) | 2'911.00 (0-55'130.00) | 926.67 (130.00-3'580.00) | 1'218.00 (110.00-7'540.00) | 0.941 | 0.770 | 0.828 | 0.907 |
| Creatinine baseline 1 year after transplantation, μmol/L* | 112.38 (52.00-271.00) | 112.33 (61.33-271.00) | 120.04 (78.33-194.00) | 107.91 (52.00-167.67) | 0.743 | 0.453 | 0.865 | 0.599 |
| Proteinuria baseline 1 year after transplantation, mg/day* | 196.87 (0-1'693.33) | 180.08 (0-1'066.67) | 284.44 (0-1'693.33) | 190.98 (0-576.67) | 0.502 | 0.655 | 0.244 | 0.751 |
| Creatinine: Worsening between first and third baseline, n (%) | 27 (40) | 11 (26) | 5 (56) | 11 (65) | 0.014* | 0.118 | 0.008* | 0.692 |
| Proteinuria: Worsening between first and third baseline, n (%) | 20 (29) | 12 (29) | 5 (56) | 3 (18) | 0.157 | 0.140 | 0.516 | 0.078 |
| Creatinine: Stable between first and third baseline, n (%) | 25 (37) | 20 (48) | 2 (22) | 3 (18) | 0.067 | 0.268 | 0.042* | 1.000 |
| Proteinuria Stable between first and third baseline, n (%) | 12 (18) | 7 (17) | 2 (22) | 3 (18) | 0.901 | 0.651 | 1.000 | 1.000 |
| Creatinine: Improving between first and third baseline, n (%) | 13 (19) | 8 (19) | 2 (22) | 3 (18) | 1.000 | 1.000 | 1.000 | 1.000 |
| Proteinuria: Improving between first and third baseline, n (%) | 18 (26) | 11 (26) | 2 (22) | 5 (29) | 1.000 | 1.000 | 1.000 | 1.000 |
| DGF, n (%) | 15 (22) | 11 (26) | 1 (11) | 3 (18) | 0.702 | 0.666 | 0.737 | 1.000 |
| Dialysis treatment after transplantation, n (%) | 17 (25) | 11 (26) | 2 (22) | 4 (24) | 1.000 | 1.000 | 1.000 | 1.000 |
| De-novo DSA, n (%) | 28 (41) | 11 (26) | 8 (89) | 9 (53) | 0.001* | 0.001* | 0.070 | 0.098 |
| **Viral infections** |  |  |  |  |  |  |  |  |
| BKV replication at any time after transplantation, n (%) | 28 (41) | 16 (38) | 3 (33) | 9 (53) | 0.559 | 1.000 | 0.386 | 0.429 |
| ·       BKV replication over detection limit, n (%) | 22 (32) | 11 (26) | 3 (33) | 8 (47) | 0.298 | 0.692 | 0.137 | 0.683 |
| ·       BKV positivity below detection limit, n (%) | 6 (9) | 5 (12) | 0 (0) | 1 (6) | 0.704 | 0.571 | 0.662 | 1.000 |
| BKV replication between transplantation and first biopsy, n (%) | 18 (26) | 11 (26) | 3 (33) | 4 (24) | 0.851 | 0.692 | 1.000 | 0.661 |
| BKV replication at time of first biopsy, n (%) | 10 (15) | 4 (10) | 2 (22) | 4 (24) | 0.240 | 0.284 | 0.211 | 1.000 |
| CMV replication at any time after transplantation, n (%) | 43 (63) | 27 (64) | 3 (33) | 13 (76) | 0.100 | 0.136 | 0.540 | 0.046* |
| CMV replication between transplantation and first biopsy, n (%) | 21 (31) | 12 (29) | 2 (22) | 7 (41) | 0.597 | 1.000 | 0.372 | 0.418 |
| CMV replication at time of first biopsy, n (%) | 10 (15) | 6 (14) | 0 (0) | 4 (24) | 0.316 | 0.575 | 0.453 | 0.263 |

*median (range)

**C**

|  | **Total**  n=68 | **NR/BLR**  n=42 | **ABMR**  n=9 | **TCMR**  n=17 | **P**  all groups | **P**  NR/BLR vs. ABMR | **P**  NR/BLR vs. TCMR | **P**  ABMR vs. TCMR |
| --- | --- | --- | --- | --- | --- | --- | --- | --- |
| **2. Renal biopsy** |  |  |  |  |  |  |  |  |
| Time post-1st biopsy, months* | 4 (0-25) | 6 (0-24) | 15 (0-25) | 2 (0-18) | 0.018* | 0.311 | 0.008* | 0.066 |
| ·       <6 months, n (%) | 38 (56) | 20 (48) | 3 (33) | 15 (88) | 0.004* | 0.487 | 0.007* | 0.008* |
| ·       6-12 months, n (%) | 14 (21) | 12 (29) | 1 (11) | 1 (6) | 0.123 | 0.417 | 0.084 | 1.000 |
| ·       13-25 months, n (%) | 16 (24) | 10 (24) | 5 (56) | 1 (6) | 0.020* | 0.102 | 0.151 | 0.010* |
| Indication for second biopsy |  |  |  |  |  |  |  |  |
| ·       eGFR, n (%) | 15 (22) | 7 (17) | 1 (11) | 7 (41) | 0.100 | 1.000 | 0.087 | 0.190 |
| ·       Proteinuria, n (%) | 16 (24) | 13 (31) | 2 (22) | 1 (6) | 0.092 | 0.709 | 0.048* | 0.268 |
| ·       eGFR and proteinuria, n (%) | 22 (32) | 12 (29) | 4 (44) | 6 (35) | 0.563 | 0.436 | 0.756 | 0.692 |
| ·       DSA, n (%) | 1 (1) | 0 (0) | 1 (11) | 0 (0) | 0.132 | 0.176 | 1.000 | 0.346 |
| ·       eGFR and BKV, n (%) | 0 (0) | 0 (0) | 0 (0) | 0 (0) | 1.000 | 1.000 | 1.000 | 1.000 |
| ·       Other, n (%) | 14 (21) | 10 (24) | 1 (11) | 3 (18) | 0.752 | 0.663 | 0.738 | 1.000 |
| Banff-Score, n (%) |  |  |  |  |  |  |  |  |
| ·       t-lesion ≥ 2 | 16 (24) | 4 (10) | 3 (33) | 9 (53) | 0.001* | 0.095 | 0.001* | 0.429 |
| ·       t-lesion = 1 | 20 (29) | 12 (29) | 3 (33) | 5 (29) | 1.000 | 1.000 | 1.000 | 1.000 |
| ·       i-lesion ≥ 2 | 12 (18) | 2 (5) | 2 (22) | 8 (47) | 0.001* | 0.139 | 0.000* | 0.399 |
| ·       i-lesion = 1 | 6 (9) | 2 (5) | 2 (22) | 2 (12) | 0.142 | 0.139 | 0.571 | 0.591 |
| ·       ti-lesion ≥ 2 | 20 (29) | 9 (21) | 3 (33) | 8 (47) | 0.134 | 0.424 | 0.063 | 0.683 |
| ·       ti-lesion = 1 | 14 (21) | 6 (14) | 4 (44) | 4 (24) | 0.109 | 0.061 | 0.453 | 0.382 |
| ·       ptc-lesion ≥ 2 | 8 (12) | 1 (2) | 3 (33) | 4 (24) | 0.003* | 0.015* | 0.021* | 0.661 |
| ·       ptc-lesion = 1 | 5 (7) | 0 (0) | 3 (33) | 2 (12) | 0.002* | 0.004* | 0.079 | 0.302 |
| ·       v-lesion ≥ 2 | 5 (7) | 1 (2) | 0 (0) | 4 (24) | 0.029* | 1.000 | 0.021* | 0.263 |
| ·       v-lesion = 1 | 17 (25) | 0 (0) | 5 (56) | 12 (71) | 0.000* | 0.000* | 0.000* | 0.667 |
| ·       cv-lesion ≥ 2 | 7 (10) | 4 (10) | 2 (22) | 1 (6) | 0.362 | 0.284 | 1.000 | 0.268 |
| ·       cv-lesion = 1 | 14 (21) | 7 (17) | 1 (11) | 6 (35) | 0.307 | 1.000 | 0.166 | 0.357 |
| ·       g-lesion ≥ 2 | 5 (7) | 0 (0) | 3 (33) | 2 (12) | 0.002* | 0.004* | 0.079 | 0.302 |
| ·       g-lesion = 1 | 10 (15) | 4 (10) | 4 (44) | 2 (12) | 0.041* | 0.025* | 1.000 | 0.138 |
| ·       cg-lesion ≥ 2 | 3 (4) | 1 (2) | 2 (22) | 0 (0) | 0.082 | 0.077 | 1.000 | 0.111 |
| ·       cg-lesion = 1 | 6 (9) | 2 (5) | 1 (11) | 3 (18) | 0.238 | 0.449 | 0.138 | 1.000 |
| ·       mm-lesion ≥ 2 | 3 (4) | 2 (5) | 1 (11) | 0 (0) | 0.479 | 0.449 | 1.000 | 0.346 |
| ·       mm-lesion = 1 | 5 (7) | 4 (10) | 0 (0) | 1 (6) | 1.000 | 1.000 | 1.000 | 1.000 |
| ·       ci-lesion ≥ 2 | 16 (24) | 13 (31) | 2 (22) | 1 (6) | 0.092 | 0.709 | 0.048* | 0.268 |
| ·       ci-lesion = 1 | 31 (46) | 15 (36) | 6 (67) | 10 (59) | 0.132 | 0.136 | 0.147 | 1.000 |
| ·       ct-lesion ≥ 2 | 16 (24) | 13 (31) | 2 (22) | 1 (6) | 0.092 | 0.709 | 0.048* | 0.268 |
| ·       ct-lesion = 1 | 46 (68) | 24 (57) | 7 (78) | 15 (88) | 0.059 | 0.454 | 0.033* | 0.591 |
| ·       ah-lesion ≥ 2 | 25 (37) | 14 (33) | 6 (67) | 5 (29) | 0.155 | 0.129 | 1.000 | 0.103 |
| ·       ah-lesion = 1 | 33 (49) | 21 (50) | 3 (33) | 9 (53) | 0.684 | 0.473 | 1.000 | 0.429 |
| ·       aah-lesion ≥ 2 | 9 (13) | 6 (14) | 2 (22) | 1 (6) | 0.449 | 0.619 | 0.661 | 0.268 |
| ·       aah-lesion = 1 | 12 (18) | 8 (19) | 2 (22) | 2 (12) | 0.731 | 1.000 | 0.708 | 0.591 |
| ·       cd4-lesion ≥ 2 | 9 (13) | 5 (12) | 3 (33) | 1 (6) | 0.172 | 0.137 | 0.662 | 0.104 |
| ·       cd4-lesion = 1 | 1 (1) | 0 (0) | 0 (0) | 1 (6) | 0.382 | 1.000 | 0.288 | 1.000 |
| **Immunosuppression** |  |  |  |  |  |  |  |  |
| Steroid, n (%) | 20 (29) | 1 (2) | 5 (56) | 14 (82) | 0.000* | 0.000* | 0.000* | 0.188 |
| Privigen, n (%) | 2 (3) | 0 (0) | 1 (11) | 1 (6) | 0.143 | 0.176 | 0.288 | 1.000 |
| Rituximab, n (%) | 1 (1) | 0 (0) | 0 (0) | 1 (6) | 0.382 | 1.000 | 0.288 | 1.000 |
| PEX, n (%) | 2 (3) | 0 (0) | 2 (22) | 0 (0) | 0.016* | 0.028* | 1.000 | 0.111 |
| Other, n (%) | 1 (1) | 0 (0) | 1 (11) | 0 (0) | 0.132 | 0.176 | 1.000 | 0.346 |
| **Kidney transplant function** |  |  |  |  |  |  |  |  |
| Creatinine baseline 1 year before second biopsy, μmol/L* | 176.21 (65.67-549.00) | 166.57 (65.67-472.67) | 188.59 (124.00-271.00) | 192.92 (87.67-549.00) | 0.217 | 0.061 | 0.791 | 0.339 |
| Creatinine at second biopsy, μmol/L* | 261.76 (72.00-1'090.00) | 207.1 (72.00-569.00) | 365.33 (141.00-1'090.00) | 338.76 (95.00-1'021.00) | 0.015* | 0.051 | 0.011* | 0.958 |
| Creatinine baseline 1 year after second biopsy, μmol/L* | 172.38 (61.33-476.33) | 153.26 (61.33-451.67) | 199.11 (78.33-476.33) | 202.08 (72.67-460.33) | 0.108 | 0.181 | 0.058 | 0.958 |
| Proteinuria baseline 1 year before second biopsy, mg/day* | 881.75 (0-11'563.33) | 820.82 (0-11'390.00) | 696.04 (76.67-2'720.00) | 1'131.11 (40.00-11'563.33) | 0.241 | 0.161 | 0.233 | 0.681 |
| Proteinuria at second biopsy, mg/day* | 1'255.41 (0-14'890.00) | 1'060.13 (0-7'260.00) | 914.44 (140.00-2'700.00) | 2'162.08 (0-14'890.00) | 0.329 | 0.205 | 0.403 | 0.345 |
| Proteinuria baseline 1 year after second biopsy, mg/day* | 530.35 (0-4'545.00) | 401.14 (0-3'633.33) | 1'275.74 (210.00-4'545.00) | 410.44 (0-2'330.00) | 0.011* | 0.004* | 0.813 | 0.004* |
| **Viral infections** |  |  |  |  |  |  |  |  |
| BKV replication between first and second biopsy, n (%) | 21 (31) | 11 (26) | 2 (22) | 8 (47) | 0.282 | 1.000 | 0.137 | 0.399 |
| BKV replication at time of second biopsy, n (%) | 15 (22) | 8 (19) | 2 (22) | 5 (29) | 0.762 | 1.000 | 0.491 | 1.000 |
| CMV replication between first and second biopsy, n (%) | 30 (44) | 19 (45) | 1 (11) | 10 (59) | 0.050* | 0.072 | 0.399 | 0.036* |
| CMV replication at time of second biopsy, n (%) | 8 (12) | 5 (12) | 0 (0) | 3 (18) | 0.561 | 0.571 | 0.678 | 0.529 |

*median (range)

**Supplement Table 3A-C.** Basic characteristics (A), characteristics at 1^st^ biopsy (B) and at 2^nd^ biopsy (C) of 21 KTRs with TCMR in the first indication biopsy.

**A**

|  | **Total**  n=21 | **NR/BLR**  n=12 | **ABMR**  n=3 | **TCMR**  n=6 | ***P***  *all groups* | ***P*** *NR/BLR vs. ABMR* | ***P*** *NR/BLR vs. TCMR* | ***P***  *ABMR vs. TCMR* |
| --- | --- | --- | --- | --- | --- | --- | --- | --- |
| **Recipient characteristics** |  |  |  |  |  |  |  |  |
| Recipient age, years* | 51 (30-73) | 46 (30-61) | 55 (51-70) | 56.5 (37-73) | *0.151* | *0.101* | *0.151* | *1.000* |
| Recipient, male sex, n (%) | 15 (71) | 9 (75) | 3 (100) | 3 (50) | *0.429* | *1.000* | *0.344* | *0.464* |
| Renal disease, n (%) |  |  |  |  |  |  |  |  |
| ·       Diabetic | 0 (0) | 0 (0) | 0 (0) | 0 (0) | *1.000* | *1.000* | *1.000* | *1.000* |
| ·       Hypertensive | 2 (10) | 0 (0) | 2 (67) | 0 (0) | *0.014** | *0.029** | *1.000* | *0.083* |
| ·       PKD | 2 (10) | 2 (17) | 0 (0) | 0 (0) | *0.657* | *1.000* | *0.529* | *1.000* |
| ·       Glomerular disease | 4 (19) | 2 (17) | 0 (0) | 2 (33) | *0.581* | *1.000* | *0.569* | *0.500* |
| ·       Reflux nephropathy | 2 (10) | 2 (17) | 0 (0) | 0 (0) | *0.657* | *1.000* | *0.529* | *1.000* |
| ·       Others | 7 (33) | 3 (25) | 1 (33) | 3 (50) | *0.686* | *1.000* | *0.344* | *1.000* |
| ·       Unknown | 4 (19) | 3 (25) | 0 (0) | 1 (17) | *1.000* | *1.000* | *1.000* | *1.000* |
| Living donation, n (%) | 4 (19) | 2 (17) | 0 (0) | 2 (33) | *0.581* | *1.000* | *0.569* | *0.500* |
| Deceased donation, n (%) | 17 (81) | 10 (83) | 3 (100) | 4 (67) | *0.581* | *1.000* | *0.569* | *0.500* |
| AB0-incompatible, n (%) | 3 (14) | 1 (8) | 0 (0) | 2 (33) | *0.226* | *1.000* | *0.245* | *0.500* |
| Kidney/Pancreas-transplantation, n (%) | 0 (0) | 0 (0) | 0 (0) | 0 (0) | *1.000* | *1.000* | *1.000* | *1.000* |
| Retransplantation, n (%) | 2 (10) | 1 (8) | 0 (0) | 1 (17) | *1.000* | *1.000* | *1.000* | *1.000* |
| Cold ischemia time* | 535 (195-1'000) | 593 (308-1'000) | 477 (413-540) | 432 (195-538) | *0.736* | *0.692* | *0.539* | *1.000* |
| Tumor before transplantation, n (%) | 1 (5) | 1 (8) | 0 (0) | 0 (0) | *1.000* | *1.000* | *1.000* | *1.000* |
| Tumor after transplantation, n (%) | 0 (0) | 0 (0) | 0 (0) | 0 (0) | *1.000* | *1.000* | *1.000* | *1.000* |
| **Immunosuppression** |  |  |  |  |  |  |  |  |
| ·       Tacrolismus, n (%) | 19 (90) | 10 (83) | 3 (100) | 6 (100) | *0.657* | *1.000* | *0.529* | *1.000* |
| ·       Ciclosporine, n (%) | 2 (10) | 2 (17) | 0 (0) | 0 (0) | *0.657* | *1.000* | *0.529* | *1.000* |
| ·       Cellcept (MMF), n (%) | 15 (71) | 9 (75) | 2 (67) | 4 (67) | *1.000* | *1.000* | *1.000* | *1.000* |
| ·       Myofortic (MPA), n (%) | 6 (29) | 3 (25) | 1 (33) | 2 (33) | *1.000* | *1.000* | *1.000* | *1.000* |
| **Donor characteristics** |  |  |  |  |  |  |  |  |
| Donor age, years* | 55 (23-74) | 57 (40-73) | 70 (46-74) | 50.5 (23-74) | *0.343* | *0.295* | *0.291* | *0.381* |
| Donor, male sex, n (%) | 12 (57) | 6 (50) | 3 (100) | 3 (50) | *0.399* | *0.229* | *1.000* | *0.464* |
| **Immunocompatibility** |  |  |  |  |  |  |  |  |
| Total HLA mismatches* | 7 (4-9) | 6.5 (4-9) | 6 (5-8) | 7.5 (6-9) | *0.274* | *0.840* | *0.180* | *0.262* |
| Total PIRCHE-Score* | 79.03 (16.00-233.55) | 76.52 (32.30-233.55) | 63.76 (16.00-134.97) | 101.5 (36.88-126.44) | *0.664* | *0.633* | *0.385* | *0.905* |
| ·       PIRCHE-A | 13.03 (1.00-42.87) | 14.62 (6.38-40.07) | 4.28 (1.00-42.87) | 11.75 (7.00-25.35) | *0.591* | *0.448* | *0.616* | *0.548* |
| ·       PIRCHE-B | 14 (0-53.40) | 10.43 (0-53.40) | 8 (7.00-21.03) | 17.86 (12.00-24.69) | *0.502* | *1.000* | *0.335* | *0.381* |
| ·       PIRCHE-C | 12.64 (0-75.06) | 10 (0-75.06) | 19.48 (0-43.61) | 19 (0.40-38.00) | *0.862* | *0.840* | *0.616* | *1.000* |
| ·       PIRCHE-DR | 15 (3.00-37.93) | 13.5 (7.08-37.93) | 14 (3.00-21.00) | 16.76 (7.00-31.90) | *0.623* | *0.840* | *0.437* | *0.548* |
| ·       PIRCHE-DQ | 21.04 (4.00-47.39) | 21.05 (9.73-47.39) | 8.09 (4.00-19.00) | 25.57 (8.91-37.86) | *0.107* | *0.048** | *1.000* | *0.095* |
| ·       PIRCHE HLA-I | 36 (7.09-148.23) | 34.15 (7.09-148.23) | 30.76 (9.00-107.51) | 47.94 (20.98-73.00) | *0.762* | *0.840* | *0.553* | *0.714* |
| ·       PIRCHE HLA-II | 33 (7.00-85.32) | 34.77 (17.43-85.32) | 29.09 (7.00-33.00) | 49.18 (15.91-59.90) | *0.397* | *0.295* | *0.750* | *0.262* |
| Preformed DSA, n (%) | 6 (29) | 3 (25) | 1 (33) | 2 (33) | *1.000* | *1.000* | *1.000* | *1.000* |

*median (range)

**B**

|  | **Total**  n=21 | **NR/BLR**  n=12 | **ABMR**  n=3 | **TCMR**  n=6 | ***P***  *all groups* | ***P*** *NR/BLR vs. ABMR* | ***P*** *NR/BLR vs. TCMR* | ***P***  *ABMR vs. TCMR* |
| --- | --- | --- | --- | --- | --- | --- | --- | --- |
| **1. Renal biopsy** |  |  |  |  |  |  |  |  |
| Time post-transplant, months* | 2 (0-46) | 1.5 (0-28) | 3 (2-46) | 3.5 (0-8) | *0.500* | *0.233* | *1.000* | *0.548* |
| ·       <6 months, n (%) | 14 (67) | 9 (75) | 2 (67) | 3 (50) | *0.686* | *1.000* | *0.344* | *1.000* |
| ·       6-12 months, n (%) | 4 (19) | 1 (8) | 0 (0) | 3 (50) | *0.132* | *1.000* | *0.083* | *0.464* |
| ·       13-60 months, n (%) | 3 (14) | 2 (17) | 1 (33) | 0 (0) | *0.374* | *0.516* | *0.529* | *0.333* |
| ·       >60 months, n (%) | 0 (0) | 0 (0) | 0 (0) | 0 (0) | *1.000* | *1.000* | *1.000* | *1.000* |
| Indication for 1. biopsy |  |  |  |  |  |  |  |  |
| ·       eGFR, n (%) | 8 (38) | 6 (50) | 0 (0) | 2 (33) | *0.340* | *0.229* | *0.638* | *0.500* |
| ·       Proteinuria, n (%) | 2 (10) | 1 (8) | 0 (0) | 1 (17) | *1.000* | *1.000* | *1.000* | *1.000* |
| ·       eGFR and proteinuria, n (%) | 7 (33) | 3 (25) | 2 (67) | 2 (33) | *0.470* | *0.242* | *1.000* | *0.524* |
| ·       eGFR and BKV, n (%) | 3 (14) | 1 (8) | 1 (33) | 1 (17) | *0.537* | *0.371* | *1.000* | *1.000* |
| ·       DSA n (%) | 0 (0) | 0 (0) | 0 (0) | 0 (0) | *1.000* | *1.000* | *1.000* | *1.000* |
| ·       Other, n (%) | 1 (5) | 1 (8) | 0 (0) | 0 (0) | *1.000* | *1.000* | *1.000* | *1.000* |
| Banff-Score, n (%) |  |  |  |  |  |  |  |  |
| ·       t-lesion ≥ 2 | 5 (24) | 3 (25) | 0 (0) | 2 (33) | *0.805* | *1.000* | *1.000* | *0.500* |
| ·       t-lesion = 1 | 9 (43) | 4 (33) | 2 (67) | 3 (50) | *0.576* | *0.525* | *0.627* | *1.000* |
| ·       i-lesion ≥ 2 | 4 (19) | 0 (0) | 1 (33) | 3 (50) | *0.023** | *0.200* | *0.025** | *1.000* |
| ·       i-lesion = 1 | 3 (14) | 3 (25) | 0 (0) | 0 (0) | *0.702* | *1.000* | *0.515* | *1.000* |
| ·       ti-lesion ≥ 2 | 6 (29) | 1 (8) | 1 (33) | 4 (67) | *0.027** | *0.371* | *0.022** | *0.524* |
| ·       ti-lesion = 1 | 5 (24) | 3 (25) | 2 (67) | 0 (0) | *0.081* | *0.242* | *0.515* | *0.083* |
| ·       ptc-lesion ≥ 2 | 3 (14) | 0 (0) | 0 (0) | 3 (50) | *0.029** | *1.000* | *0.025** | *0.464* |
| ·       ptc-lesion = 1 | 1 (5) | 0 (0) | 0 (0) | 1 (17) | *0.429* | *1.000* | *0.333* | *1.000* |
| ·       v-lesion ≥ 2 | 2 (10) | 1 (8) | 0 (0) | 1 (17) | *1.000* | *1.000* | *1.000* | *1.000* |
| ·       v-lesion = 1 | 16 (76) | 10 (83) | 1 (33) | 5 (83) | *0.213* | *0.154* | *1.000* | *0.226* |
| ·       cv-lesion ≥ 2 | 2 (10) | 0 (0) | 1 (33) | 1 (17) | *0.171* | *0.200* | *0.333* | *1.000* |
| ·       cv-lesion = 1 | 7 (33) | 5 (42) | 0 (0) | 2 (33) | *0.572* | *0.505* | *1.000* | *0.500* |
| ·       g-lesion ≥ 2 | 0 (0) | 0 (0) | 0 (0) | 0 (0) | *1.000* | *1.000* | *1.000* | *1.000* |
| ·       g-lesion = 1 | 3 (14) | 2 (17) | 1 (33) | 0 (0) | *0.374* | *0.516* | *0.529* | *0.333* |
| ·       cg-lesion ≥ 2 | 0 (0) | 0 (0) | 0 (0) | 0 (0) | *1.000* | *1.000* | *1.000* | *1.000* |
| ·       cg-lesion = 1 | 3 (14) | 1 (8) | 1 (33) | 1 (17) | *0.537* | *0.371* | *1.000* | *1.000* |
| ·       mm-lesion ≥ 2 | 0 (0) | 0 (0) | 0 (0) | 0 (0) | *1.000* | *1.000* | *1.000* | *1.000* |
| ·       mm-lesion = 1 | 1 (5) | 0 (0) | 1 (33) | 0 (0) | *0.143* | *0.200* | *1.000* | *0.333* |
| ·       ci-lesion ≥ 2 | 2 (10) | 1 (8) | 0 (0) | 1 (17) | *1.000* | *1.000* | *1.000* | *1.000* |
| ·       ci-lesion = 1 | 7 (33) | 3 (25) | 2 (67) | 2 (33) | *0.470* | *0.242* | *1.000* | *0.524* |
| ·       ct-lesion ≥ 2 | 2 (10) | 1 (8) | 0 (0) | 1 (17) | *1.000* | *1.000* | *1.000* | *1.000* |
| ·       ct-lesion = 1 | 10 (48) | 5 (42) | 2 (67) | 3 (50) | *0.843* | *0.569* | *1.000* | *1.000* |
| ·       ah-lesion ≥ 2 | 2 (10) | 1 (8) | 0 (0) | 1 (17) | *1.000* | *1.000* | *1.000* | *1.000* |
| ·       ah-lesion = 1 | 13 (62) | 9 (75) | 2 (67) | 2 (33) | *0.207* | *1.000* | *0.141* | *0.524* |
| ·       aah-lesion ≥ 2 | 1 (5) | 1 (8) | 0 (0) | 0 (0) | *1.000* | *1.000* | *1.000* | *1.000* |
| ·       aah-lesion = 1 | 1 (5) | 1 (8) | 0 (0) | 0 (0) | *1.000* | *1.000* | *1.000* | *1.000* |
| ·       cd4-lesion ≥ 2 | 1 (5) | 1 (8) | 0 (0) | 0 (0) | *1.000* | *1.000* | *1.000* | *1.000* |
| ·       cd4-lesion = 1 | 1 (5) | 1 (8) | 0 (0) | 0 (0) | *1.000* | *1.000* | *1.000* | *1.000* |
| **Immunosuppression** |  |  |  |  |  |  |  |  |
| Steroid, n (%) | 21 (100) | 12 (100) | 3 (100) | 6 (100) | *1.000* | *1.000* | *1.000* | *1.000* |
| Privigen, n (%) | 2 (10) | 1 (8) | 0 (0) | 1 (17) | *1.000* | *1.000* | *1.000* | *1.000* |
| Rituximab, n (%) | 0 (0) | 0 (0) | 0 (0) | 0 (0) | *1.000* | *1.000* | *1.000* | *1.000* |
| PEX, n (%) | 2 (10) | 2 (17) | 0 (0) | 0 (0) | *0.657* | *1.000* | *0.529* | *1.000* |
| Other, n (%) | 1 (5) | 0 (0) | 0 (0) | 1 (17) | *0.429* | *1.000* | *0.333* | *1.000* |
| **Kidney transplant function** |  |  |  |  |  |  |  |  |
| Creatinine baseline 1 year before biopsy, μmol/L* | 180.62 (58.33-452.67) | 217.14 (100.33-452.67) | 159.67 (133.00-196.00) | 118.06 (58.33-199.33) | *0.382* | *1.000* | *0.250* | *0.262* |
| Creatinine at biopsy, μmol/L* | 267.83 (89.00-527.00) | 268.83 (104.00-527.00) | 265.33 (202.00-382.00) | 267.08 (89.00-507.00) | *0.894* | *1.000* | *0.682* | *1.000* |
| Proteinuria baseline 1 year before biopsy, mg/day* | 295.88 (30.00-1'113.33) | 330.33 (36.67-1'113.33) | 260 (130.00-486.67) | 236.67 (30.00-623.33) | *0.860* | *0.692* | *0.839* | *1.000* |
| Proteinuria at biopsy, mg/day* | 571.58 (70.00-3'310.00) | 474.17 (70.00-1'990.00) | 373.33 (240.00-460.00) | 1'012.50 (90.00-3'310.00) | *0.944* | *0.840* | *0.862* | *1.000* |
| Creatinine baseline 1 year after transplantation, μmol/L* | 132.59 (53.67-263.67) | 135.53 (53.67-263.67) | 176.56 (134.67-199.00) | 104.72 (54.33-150.67) | *0.119* | *0.136* | *0.494* | *0.048** |
| Proteinuria baseline 1 year after transplantation, mg/day* | 168.41 (0-673.33) | 146.39 (0-673.33) | 122.22 (0-246.67) | 235.56 (0-666.67) | *0.876* | *1.000* | *0.682* | *0.714* |
| Creatinine: Worsening between first and third baseline, n (%) | 9 (43) | 5 (42) | 2 (67) | 2 (33) | *0.697* | *0.569* | *1.000* | *0.524* |
| Proteinuria: Worsening between first and third baseline, n (%) | 7 (33) | 4 (33) | 1 (33) | 2 (33) | *1.000* | *1.000* | *1.000* | *1.000* |
| Creatinine: Stable between first and third baseline, n (%) | 8 (38) | 4 (33) | 1 (33) | 3 (50) | *0.825* | *1.000* | *0.627* | *1.000* |
| Proteinuria Stable between first and third baseline, n (%) | 2 (10) | 1 (8) | 1 (33) | 0 (0) | *0.343* | *0.371* | *1.000* | *0.333* |
| Creatinine: Improving between first and third baseline, n (%) | 4 (19) | 3 (25) | 0 (0) | 1 (17) | *1.000* | *1.000* | *1.000* | *1.000* |
| Proteinuria: Improving between first and third baseline, n (%) | 8 (38) | 5 (42) | 1 (33) | 2 (33) | *1.000* | *1.000* | *1.000* | *1.000* |
| DGF, n (%) | 5 (24) | 4 (33) | 0 (0) | 1 (17) | *0.643* | *0.516* | *0.615* | *1.000* |
| Dialysis treatment after transplantation, n (%) | 6 (29) | 4 (33) | 1 (33) | 1 (17) | *0.818* | *1.000* | *0.615* | *1.000* |
| De-novo DSA, n (%) | 8 (38) | 4 (33) | 2 (67) | 2 (33) | *0.679* | *0.525* | *1.000* | *0.524* |
| **Viral infections** |  |  |  |  |  |  |  |  |
| BKV replication at any time after transplantation, n (%) | 11 (52) | 7 (58) | 2 (67) | 2 (33) | *0.590* | *1.000* | *0.620* | *0.524* |
| ·       BKV replication over detection limit, n (%) | 9 (43) | 6 (50) | 2 (67) | 1 (17) | *0.336* | *1.000* | *0.316* | *0.226* |
| ·       BKV positivity below detection limit, n (%) | 2 (10) | 1 (8) | 0 (0) | 1 (17) | *1.000* | *1.000* | *1.000* | *1.000* |
| BKV replication between transplantation and first biopsy, n (%) | 3 (14) | 1 (8) | 1 (33) | 1 (17) | *0.537* | *0.371* | *1.000* | *1.000* |
| BKV replication at time of first biopsy, n (%) | 2 (10) | 0 (0) | 1 (33) | 1 (17) | *0.171* | *0.200* | *0.333* | *1.000* |
| CMV replication at any time after transplantation, n (%) | 13 (62) | 8 (67) | 2 (67) | 3 (50) | *0.825* | *1.000* | *0.627* | *1.000* |
| CMV replication between transplantation and first biopsy, n (%) | 7 (33) | 3 (25) | 2 (67) | 2 (33) | *0.470* | *0.242* | *1.000* | *0.524* |
| CMV replication at time of first biopsy, n (%) | 3 (14) | 3 (25) | 0 (0) | 0 (0) | *0.702* | *1.000* | *0.515* | *1.000* |

*median (range)

**C**

|  | **Total**  n=21 | **NR/BLR**  n=12 | **ABMR**  n=3 | **TCMR**  n=6 | ***P***  *all groups* | ***P***  *NR/BLR vs. ABMR* | ***P*** *NR/BLR vs. TCMR* | ***P***  *ABMR vs. TCMR* |
| --- | --- | --- | --- | --- | --- | --- | --- | --- |
| **2. Renal biopsy** |  |  |  |  |  |  |  |  |
| Time post-1st biopsy, months* | 1 (0-14) | 1.5 (0-14) | 1 (0-1) | 0.5 (0-5) | 0.647 | 0.448 | 0.616 | 0.905 |
| ·       <6 months, n (%) | 19 (90) | 10 (83) | 3 (100) | 6 (100) | 0.657 | 1.000 | 0.529 | 1.000 |
| ·       6-12 months, n (%) | 1 (5) | 1 (8) | 0 (0) | 0 (0) | 1.000 | 1.000 | 1.000 | 1.000 |
| ·       13-25 months, n (%) | 1 (5) | 1 (8) | 0 (0) | 0 (0) | 1.000 | 1.000 | 1.000 | 1.000 |
| Indication for second biopsy |  |  |  |  |  |  |  |  |
| ·       Inadequate treatment response, n (%) | 14 (67) | 6 (50) | 3 (100) | 5 (83) | 0.223 | 0.229 | 0.316 | 1.000 |
| ·       Other, n (%) | 7 (33) | 6 (50) | 0 (0) | 1 (17) | 0.223 | 0.229 | 0.316 | 1.000 |
| Banff-Score, n (%) |  |  |  |  |  |  |  |  |
| ·       t-lesion ≥ 2 | 9 (43) | 3 (25) | 2 (67) | 4 (67) | 0.150 | 0.242 | 0.141 | 1.000 |
| ·       t-lesion = 1 | 6 (29) | 4 (33) | 1 (33) | 1 (17) | 0.818 | 1.000 | 0.615 | 1.000 |
| ·       i-lesion ≥ 2 | 3 (14) | 0 (0) | 1 (33) | 2 (33) | 0.090 | 0.200 | 0.098 | 1.000 |
| ·       i-lesion = 1 | 3 (14) | 1 (8) | 2 (67) | 0 (0) | 0.056 | 0.081 | 1.000 | 0.083 |
| ·       ti-lesion ≥ 2 | 5 (24) | 1 (8) | 1 (33) | 3 (50) | 0.116 | 0.371 | 0.083 | 1.000 |
| ·       ti-lesion = 1 | 6 (29) | 3 (25) | 2 (67) | 1 (17) | 0.348 | 0.242 | 1.000 | 0.226 |
| ·       ptc-lesion ≥ 2 | 7 (33) | 1 (8) | 3 (100) | 3 (50) | 0.004* | 0.009* | 0.083 | 0.464 |
| ·       ptc-lesion = 1 | 0 (0) | 0 (0) | 0 (0) | 0 (0) | 1.000 | 1.000 | 1.000 | 1.000 |
| ·       v-lesion ≥ 2 | 1 (5) | 0 (0) | 0 (0) | 1 (17) | 0.429 | 1.000 | 0.333 | 1.000 |
| ·       v-lesion = 1 | 5 (24) | 1 (8) | 2 (67) | 2 (33) | 0.048* | 0.081 | 0.245 | 0.524 |
| ·       cv-lesion ≥ 2 | 4 (19) | 1 (8) | 2 (67) | 1 (17) | 0.092 | 0.081 | 1.000 | 0.226 |
| ·       cv-lesion = 1 | 8 (38) | 4 (33) | 1 (33) | 3 (50) | 0.825 | 1.000 | 0.627 | 1.000 |
| ·       g-lesion ≥ 2 | 1 (5) | 0 (0) | 1 (33) | 0 (0) | 0.143 | 0.200 | 1.000 | 0.333 |
| ·       g-lesion = 1 | 6 (29) | 2 (17) | 2 (67) | 2 (33) | 0.202 | 0.154 | 0.569 | 0.524 |
| ·       cg-lesion ≥ 2 | 1 (5) | 1 (8) | 0 (0) | 0 (0) | 1.000 | 1.000 | 1.000 | 1.000 |
| ·       cg-lesion = 1 | 1 (5) | 0 (0) | 0 (0) | 1 (17) | 0.429 | 1.000 | 0.333 | 1.000 |
| ·       mm-lesion ≥ 2 | 1 (5) | 0 (0) | 1 (33) | 0 (0) | 0.143 | 0.200 | 1.000 | 0.333 |
| ·       mm-lesion = 1 | 0 (0) | 0 (0) | 0 (0) | 0 (0) | 1.000 | 1.000 | 1.000 | 1.000 |
| ·       ci-lesion ≥ 2 | 4 (19) | 2 (17) | 1 (33) | 1 (17) | 0.779 | 0.516 | 1.000 | 1.000 |
| ·       ci-lesion = 1 | 11 (52) | 4 (33) | 2 (67) | 5 (83) | 0.139 | 0.525 | 0.131 | 1.000 |
| ·       ct-lesion ≥ 2 | 4 (19) | 2 (17) | 1 (33) | 1 (17) | 0.779 | 0.516 | 1.000 | 1.000 |
| ·       ct-lesion = 1 | 15 (71) | 8 (67) | 2 (67) | 5 (83) | 0.818 | 1.000 | 0.615 | 1.000 |
| ·       ah-lesion ≥ 2 | 7 (33) | 3 (25) | 3 (100) | 1 (17) | 0.043* | 0.044* | 1.000 | 0.048* |
| ·       ah-lesion = 1 | 8 (38) | 6 (50) | 0 (0) | 2 (33) | 0.340 | 0.229 | 0.638 | 0.500 |
| ·       aah-lesion ≥ 2 | 2 (10) | 2 (17) | 0 (0) | 0 (0) | 0.657 | 1.000 | 0.529 | 1.000 |
| ·       aah-lesion = 1 | 2 (10) | 1 (8) | 1 (33) | 0 (0) | 0.343 | 0.371 | 1.000 | 0.333 |
| ·       cd4-lesion ≥ 2 | 3 (14) | 1 (8) | 1 (33) | 1 (17) | 0.537 | 0.371 | 1.000 | 1.000 |
| ·       cd4-lesion = 1 | 1 (5) | 1 (8) | 0 (0) | 0 (0) | 1.000 | 1.000 | 1.000 | 1.000 |
| **Immunosuppression** |  |  |  |  |  |  |  |  |
| Steroid, n (%) | 6 (29) | 0 (0) | 2 (67) | 4 (67) | 0.002* | 0.029* | 0.005* | 1.000 |
| Privigen, n (%) | 0 (0) | 0 (0) | 0 (0) | 0 (0) | 1.000 | 1.000 | 1.000 | 1.000 |
| Rituximab, n (%) | 0 (0) | 0 (0) | 0 (0) | 0 (0) | 1.000 | 1.000 | 1.000 | 1.000 |
| PEX, n (%) | 0 (0) | 0 (0) | 0 (0) | 0 (0) | 1.000 | 1.000 | 1.000 | 1.000 |
| Other, n (%) | 2 (10) | 0 (0) | 1 (33) | 1 (17) | 0.171 | 0.200 | 0.333 | 1.000 |
| **Kidney transplant function** |  |  |  |  |  |  |  |  |
| Creatinine baseline 1 year before second biopsy, μmol/L* | 187.08 (82.33-435.00) | 165.17 (98.33-273.00) | 279.78 (173.67-435.00) | 184.55 (82.33-432.33) | 0.150 | 0.070 | 0.682 | 0.167 |
| Creatinine at second biopsy, μmol/L* | 249.55 (106.00-596.00) | 226.33 (106.00-596.00) | 354.67 (224.00-492.00) | 242.2 (128.00-388.00) | 0.194 | 0.365 | 0.721 | 0.393 |
| Creatinine baseline 1 year after second biopsy, μmol/L* | 189.25 (54.33-438.67) | 180.97 (100.67-321.67) | 292.11 (143.00-438.67) | 154.39 (54.33-341.67) | 0.274 | 0.692 | 0.385 | 0.167 |
| Proteinuria baseline 1 year before second biopsy, mg/day* | 414.63 (0-2'066.67) | 365.83 (90.00-1'360.00) | 277.78 (166.67-376.67) | 746.67 (0-2'066.67) | 0.837 | 0.536 | 1.000 | 1.000 |
| Proteinuria at second biopsy, mg/day* | 359.44 (90.00-1'260.00) | 351.67 (90.00-1'260.00) | 313.33 (190.00-400.00) | 436.67 (400.00-460.00) | 0.188 | 0.536 | 0.136 | 0.100 |
| Proteinuria baseline 1 year after second biopsy, mg/day* | 383.02 (0-3'100.00) | 186.67 (43.33-533.33) | 422.22 (113.33-920.00) | 756.11 (0-3'100.00) | 0.674 | 0.448 | 0.750 | 0.714 |
| **Viral infections** |  |  |  |  |  |  |  |  |
| BKV replication between first and second biopsy, n (%) | 10 (48) | 6 (50) | 2 (67) | 2 (33) | 0.708 | 1.000 | 0.638 | 0.524 |
| BKV replication at time of second biopsy, n (%) | 5 (24) | 2 (17) | 2 (67) | 1 (17) | 0.213 | 0.154 | 1.000 | 0.226 |
| CMV replication between first and second biopsy, n (%) | 9 (43) | 8 (67) | 0 (0) | 1 (17) | 0.036* | 0.077 | 0.131 | 1.000 |
| CMV replication at time of second biopsy, n (%) | 3 (14) | 3 (25) | 0 (0) | 0 (0) | 0.702 | 1.000 | 0.515 | 1.000 |

*median (range)

**Supplement Table 4A-C.** Basic characteristics (A), characteristics at 1^st^ biopsy (B) and at 2^nd^ biopsy (C) of 30 KTRs with TCMR in the first indication biopsy.

**A**

|  | **Total**  n=30 | **Deteriorating MVI "+"**  n=7 | **Stable MVI "0"**  n=12 | **Improving MVI "-"** n=11 | ***P***  *all groups* | ***P***  *+ vs. 0* | ***P***  *+ vs. -* | ***P***  *0 vs. -* |
| --- | --- | --- | --- | --- | --- | --- | --- | --- |
| **Recipient characteristics** |  |  |  |  |  |  |  |  |
| Recipient age, years* | 48.5 (18-74) | 43 (18-67) | 41 (21-68) | 57 (33-74) | *0.087* | *1.000* | *0.179* | *0.027** |
| Recipient, male sex, n (%) | 21 (70) | 5 (71) | 10 (83) | 6 (55) | *0.307* | *0.603* | *0.637* | *0.193* |
| Renal disease, n (%) |  |  |  |  |  |  |  |  |
| ·       Diabetic | 2 (7) | 0 (0) | 1 (8) | 1 (9) | *1.000* | *1.000* | *1.000* | *1.000* |
| ·       Hypertensive | 1 (3) | 1 (14) | 0 (0) | 0 (0) | *0.233* | *0.368* | *0.389* | *1.000* |
| ·       PKD | 5 (17) | 0 (0) | 1 (8) | 4 (36) | *0.137* | *1.000* | *0.119* | *0.155* |
| ·       Glomerular disease | 8 (27) | 1 (14) | 3 (25) | 4 (36) | *0.674* | *1.000* | *0.596* | *0.667* |
| ·       Reflux nephropathy | 3 (10) | 1 (14) | 2 (17) | 0 (0) | *0.431* | *1.000* | *0.389* | *0.478* |
| ·       Others | 6 (20) | 1 (14) | 4 (33) | 1 (9) | *0.445* | *0.603* | *1.000* | *0.317* |
| ·       Unknown | 5 (17) | 3 (43) | 1 (8) | 1 (9) | *0.202* | *0.117* | *0.245* | *1.000* |
| Living donation, n (%) | 14 (47) | 3 (43) | 5 (42) | 6 (55) | *0.897* | *1.000* | *1.000* | *0.684* |
| Deceased donation, n (%) | 16 (53) | 4 (57) | 7 (58) | 5 (45) | *0.897* | *1.000* | *1.000* | *0.684* |
| AB0-incompatible, n (%) | 0 (0) | 0 (0) | 0 (0) | 0 (0) | *1.000* | *1.000* | *1.000* | *1.000* |
| Kidney/Pancreas-transplantation, n (%) | 1 (3) | 0 (0) | 0 (0) | 1 (9) | *0.600* | *1.000* | *1.000* | *0.478* |
| Retransplantation, n (%) | 5 (17) | 2 (29) | 3 (25) | 0 (0) | *0.202* | *1.000* | *0.137* | *0.217* |
| Cold ischemia time* | 556 (276-847) | 564 (414-735) | 476 (276-698) | 662 (489-847) | *0.205* | *0.412* | *0.556* | *0.106* |
| Tumor before transplantation, n (%) | 1 (3) | 0 (0) | 0 (0) | 1 (9) | *0.600* | *1.000* | *1.000* | *0.478* |
| Tumor after transplantation, n (%) | 1 (3) | 0 (0) | 0 (0) | 1 (9) | *0.600* | *1.000* | *1.000* | *0.478* |
| **Immunosuppression** |  |  |  |  |  |  |  |  |
| ·       Tacrolismus, n (%) | 21 (70) | 3 (43) | 10 (83) | 8 (73) | *0.233* | *0.129* | *0.332* | *0.640* |
| ·       Ciclosporine, n (%) | 9 (30) | 4 (57) | 2 (17) | 3 (27) | *0.233* | *0.129* | *0.332* | *0.640* |
| ·       Cellcept (MMF), n (%) | 24 (80) | 5 (71) | 9 (75) | 10 (91) | *0.601* | *1.000* | *0.528* | *0.590* |
| ·       Myofortic (MPA), n (%) | 6 (20) | 2 (29) | 3 (25) | 1 (9) | *0.601* | *1.000* | *0.528* | *0.590* |
| **Donor characteristics** |  |  |  |  |  |  |  |  |
| Donor age, years* | 53 (31-76) | 50 (33-62) | 53 (39-69) | 56 (31-76) | *0.807* | *0.773* | *0.536* | *0.786* |
| Donor, male sex, n (%) | 12 (40) | 4 (57) | 5 (42) | 3 (27) | *0.456* | *0.650* | *0.332* | *0.667* |
| **Immunocompatibility** |  |  |  |  |  |  |  |  |
| Total HLA mismatches* | 7 (3-10) | 6 (3-10) | 6.5 (3-10) | 8 (4-10) | *0.717* | *0.902* | *0.536* | *0.525* |
| Total PIRCHE-Score* | 79.99 (25.37-200.32) | 128.38 (47.25-192.60) | 79.99 (25.37-180.27) | 70.75 (30.00-200.32) | *0.293* | *0.227* | *0.151* | *0.833* |
| ·       PIRCHE-A | 18.13 (0-55.68) | 24.22 (1.08-40.84) | 15.72 (0-55.68) | 20.25 (0-52.07) | *0.814* | *0.536* | *0.724* | *0.880* |
| ·       PIRCHE-B | 13.09 (0.28-42.77) | 17.75 (2.11-34.59) | 12.26 (0.28-22.98) | 15.09 (5.67-42.77) | *0.154* | *0.068* | *0.425* | *0.260* |
| ·       PIRCHE-C | 12.72 (0-49.37) | 19.7 (1.39-49.37) | 13.14 (0-43.85) | 11 (0-23.46) | *0.268* | *0.340* | *0.151* | *0.379* |
| ·       PIRCHE-DR | 15.01 (0-42.00) | 21.05 (0-42.00) | 17.05 (6.78-39.99) | 11.26 (1.00-39.37) | *0.401* | *0.650* | *0.246* | *0.347* |
| ·       PIRCHE-DQ | 21.5 (0-65.58) | 28.15 (10.98-65.58) | 22.09 (0-61.04) | 18.21 (0.02-43.41) | *0.248* | *0.167* | *0.151* | *0.740* |
| ·       PIRCHE HLA-I | 47.99 (4.58-118.30) | 57 (4.58-107.96) | 39.92 (11.62-91.28) | 46.43 (14.04-118.30) | *0.243* | *0.142* | *0.179* | *0.695* |
| ·       PIRCHE HLA-II | 36.5 (6.00-101.03) | 70 (14.00-86.63) | 40.15 (13.76-101.03) | 30.2 (6.00-82.78) | *0.196* | *0.167* | *0.104* | *0.566* |
| Preformed DSA, n (%) | 17 (57) | 3 (43) | 8 (67) | 6 (55) | *0.653* | *0.377* | *1.000* | *0.680* |

*median (range)

**B**

|  | **Total**  n=30 | **Deteriorating MVI "+"**  n=7 | **Stable MVI "0"**  n=12 | **Improving MVI "-"** n=11 | ***P***  *all groups* | ***P***  *+ vs. 0* | ***P***  *+ vs. -* | ***P***  *0 vs. -* |
| --- | --- | --- | --- | --- | --- | --- | --- | --- |
| **1. Renal biopsy** |  |  |  |  |  |  |  |  |
| Time post-transplant, months* | 36.5 (0-110) | 36 (0-108) | 30 (0-110) | 46 (0-75) | *0.826* | *0.711* | *1.000* | *0.566* |
| ·       <6 months, n (%) | 8 (27) | 2 (29) | 4 (33) | 2 (18) | *0.870* | *1.000* | *1.000* | *0.640* |
| ·       6-12 months, n (%) | 1 (3) | 0 (0) | 0 (0) | 1 (9) | *0.600* | *1.000* | *1.000* | *0.478* |
| ·       13-60 months, n (%) | 14 (47) | 3 (43) | 6 (50) | 5 (45) | *1.000* | *1.000* | *1.000* | *1.000* |
| ·       >60 months, n (%) | 7 (23) | 2 (29) | 2 (17) | 3 (27) | *0.750* | *0.603* | *1.000* | *0.640* |
| Indication for 1. biopsy |  |  |  |  |  |  |  |  |
| ·       eGFR, n (%) | 6 (20) | 0 (0) | 3 (25) | 3 (27) | *0.381* | *0.263* | *0.245* | *1.000* |
| ·       Proteinuria, n (%) | 9 (30) | 2 (29) | 5 (42) | 2 (18) | *0.538* | *0.656* | *1.000* | *0.371* |
| ·       eGFR and proteinuria, n (%) | 10 (33) | 3 (43) | 3 (25) | 4 (36) | *0.699* | *0.617* | *1.000* | *0.667* |
| ·       DSA, n (%) | 5 (17) | 2 (29) | 1 (8) | 2 (18) | *0.596* | *0.523* | *1.000* | *0.590* |
| Banff-Score, n (%) |  |  |  |  |  |  |  |  |
| ·       t-lesion ≥ 2 | 6 (20) | 0 (0) | 4 (33) | 2 (18) | *0.273* | *0.245* | *0.497* | *0.640* |
| ·       t-lesion = 1 | 16 (53) | 3 (43) | 6 (50) | 7 (64) | *0.721* | *1.000* | *0.630* | *0.680* |
| ·       i-lesion ≥ 2 | 3 (10) | 0 (0) | 3 (25) | 0 (0) | *0.103* | *0.263* | *1.000* | *0.217* |
| ·       i-lesion = 1 | 6 (20) | 3 (43) | 2 (17) | 1 (9) | *0.227* | *0.305* | *0.245* | *1.000* |
| ·       ti-lesion ≥ 2 | 6 (20) | 1 (14) | 3 (25) | 2 (18) | *1.000* | *1.000* | *1.000* | *1.000* |
| ·       ti-lesion = 1 | 11 (37) | 4 (57) | 5 (42) | 2 (18) | *0.214* | *0.650* | *0.141* | *0.371* |
| ·       ptc-lesion ≥ 2 | 24 (80) | 5 (71) | 9 (75) | 10 (91) | *0.601* | *1.000* | *0.528* | *0.590* |
| ·       ptc-lesion = 1 | 2 (7) | 1 (14) | 0 (0) | 1 (9) | *0.503* | *0.368* | *1.000* | *0.478* |
| ·       v-lesion ≥ 2 | 0 (0) | 0 (0) | 0 (0) | 0 (0) | *1.000* | *1.000* | *1.000* | *1.000* |
| ·       v-lesion = 1 | 9 (30) | 2 (29) | 4 (33) | 3 (27) | *1.000* | *1.000* | *1.000* | *1.000* |
| ·       cv-lesion ≥ 2 | 1 (3) | 0 (0) | 1 (8) | 0 (0) | *1.000* | *1.000* | *1.000* | *1.000* |
| ·       cv-lesion = 1 | 5 (17) | 1 (14) | 3 (25) | 1 (9) | *0.822* | *1.000* | *1.000* | *0.590* |
| ·       g-lesion ≥ 2 | 17 (57) | 3 (43) | 5 (42) | 9 (82) | *0.101* | *1.000* | *0.141* | *0.089* |
| ·       g-lesion = 1 | 8 (27) | 3 (43) | 4 (33) | 1 (9) | *0.283* | *1.000* | *0.245* | *0.317* |
| ·       cg-lesion ≥ 2 | 9 (30) | 2 (29) | 3 (25) | 4 (36) | *0.880* | *1.000* | *1.000* | *0.667* |
| ·       cg-lesion = 1 | 6 (20) | 2 (29) | 1 (8) | 3 (27) | *0.515* | *0.523* | *1.000* | *0.317* |
| ·       mm-lesion ≥ 2 | 1 (3) | 1 (14) | 0 (0) | 0 (0) | *0.233* | *0.368* | *0.389* | *1.000* |
| ·       mm-lesion = 1 | 2 (7) | 0 (0) | 1 (8) | 1 (9) | *1.000* | *1.000* | *1.000* | *1.000* |
| ·       ci-lesion ≥ 2 | 3 (10) | 1 (14) | 0 (0) | 2 (18) | *0.317* | *0.368* | *1.000* | *0.217* |
| ·       ci-lesion = 1 | 16 (53) | 3 (43) | 10 (83) | 3 (27) | *0.022** | *0.129* | *0.627* | *0.012** |
| ·       ct-lesion ≥ 2 | 3 (10) | 1 (14) | 0 (0) | 2 (18) | *0.317* | *0.368* | *1.000* | *0.217* |
| ·       ct-lesion = 1 | 23 (77) | 4 (57) | 12 (100) | 7 (64) | *0.033** | *0.036** | *1.000* | *0.037** |
| ·       ah-lesion ≥ 2 | 15 (50) | 2 (29) | 7 (58) | 6 (55) | *0.524* | *0.350* | *0.367* | *1.000* |
| ·       ah-lesion = 1 | 9 (30) | 2 (29) | 4 (33) | 3 (27) | *1.000* | *1.000* | *1.000* | *1.000* |
| ·       aah-lesion ≥ 2 | 9 (30) | 3 (43) | 4 (33) | 2 (18) | *0.605* | *1.000* | *0.326* | *0.640* |
| ·       aah-lesion = 1 | 4 (13) | 0 (0) | 1 (8) | 3 (27) | *0.324* | *1.000* | *0.263* | *0.317* |
| ·       cd4-lesion ≥ 2 | 12 (40) | 3 (43) | 6 (50) | 3 (27) | *0.573* | *1.000* | *0.627* | *0.387* |
| ·       cd4-lesion = 1 | 2 (7) | 1 (14) | 1 (8) | 0 (0) | *0.697* | *1.000* | *0.389* | *1.000* |
| **Immunosuppression** |  |  |  |  |  |  |  |  |
| Steroid, n (%) | 24 (80) | 4 (57) | 10 (83) | 10 (91) | *0.227* | *0.305* | *0.245* | *1.000* |
| Privigen, n (%) | 10 (33) | 2 (29) | 4 (33) | 4 (36) | *1.000* | *1.000* | *1.000* | *1.000* |
| Rituximab, n (%) | 2 (7) | 2 (29) | 0 (0) | 0 (0) | *0.048** | *0.123* | *0.137* | *1.000* |
| PEX, n (%) | 8 (27) | 3 (43) | 3 (25) | 2 (18) | *0.509* | *0.617* | *0.326* | *1.000* |
| Other, n (%) | 5 (17) | 1 (14) | 3 (25) | 1 (9) | *0.822* | *1.000* | *1.000* | *0.590* |
| **Kidney transplant function** |  |  |  |  |  |  |  |  |
| Creatinine baseline 1 year before biopsy, μmol/L* | 151.39 (71.00-406.33) | 164.9 (75.00-406.33) | 140.88 (71.00-331.33) | 154.26 (87.33-358.33) | *0.912* | *0.711* | *0.791* | *0.928* |
| Creatinine at biopsy, μmol/L* | 197.12 (93.00-559.00) | 177.29 (94.00-390.00) | 199.21 (93.00-421.00) | 208.5 (102.00-559.00) | *0.845* | *0.482* | *0.962* | *0.923* |
| Proteinuria baseline 1 year before biopsy, mg/day* | 248.67 (0-660.00) | 239.44 (53.33-613.33) | 246.33 (0-660.00) | 257.41 (0-580.00) | *0.947* | *0.792* | *0.864* | *1.000* |
| Proteinuria at biopsy, mg/day* | 2'160.77 (60.00-41'930.00) | 763.33 (60.00-1'980.00) | 4'097.50 (90.00-41'930.00) | 303.75 (100.00-750.00) | *0.376* | *0.682* | *0.181* | *0.343* |
| Creatinine baseline 1 year after transplantation* | 107.54 (53.00-202.33) | 104 (86.00-133.67) | 111.56 (63.33-202.33) | 105.42 (53.00-131.00) | *0.997* | *0.902* | *0.791* | *0.880* |
| Proteinuria baseline 1 year after transplantation* | 80.22 (0-260.00) | 69.52 (0-170.00) | 96.11 (0-250.00) | 69.7 (0-260.00) | *0.462* | *0.384* | *0.930* | *0.260* |
| Creatinine: Worsening between first and third baseline, n (%) | 7 (23) | 0 (0) | 5 (42) | 2 (18) | *0.129* | *0.106* | *0.497* | *0.371* |
| Proteinuria: Worsening between first and third baseline, n (%) | 10 (33) | 3 (43) | 5 (42) | 2 (18) | *0.430* | *1.000* | *0.326* | *0.371* |
| Creatinine: Stable between first and third baseline, n (%) | 16 (53) | 5 (71) | 6 (50) | 5 (45) | *0.585* | *0.633* | *0.367* | *1.000* |
| Proteinuria Stable between first and third baseline, n (%) | 5 (17) | 1 (14) | 2 (17) | 2 (18) | *1.000* | *1.000* | *1.000* | *1.000* |
| Creatinine: Improving between first and third baseline, n (%) | 4 (13) | 1 (14) | 1 (8) | 2 (18) | *0.815* | *1.000* | *1.000* | *0.590* |
| Proteinuria: Improving between first and third baseline, n (%) | 7 (23) | 1 (14) | 3 (25) | 3 (27) | *1.000* | *1.000* | *1.000* | *1.000* |
| DGF, n (%) | 4 (13) | 2 (29) | 1 (8) | 1 (9) | *0.514* | *0.523* | *0.528* | *1.000* |
| Dialysis treatment after transplantation, n (%) | 7 (23) | 2 (29) | 4 (33) | 1 (9) | *0.407* | *1.000* | *0.528* | *0.317* |
| De-novo DSA, n (%) | 22 (73) | 6 (86) | 8 (67) | 8 (73) | *0.870* | *0.603* | *1.000* | *1.000* |
| **Viral infections** |  |  |  |  |  |  |  |  |
| BKV replication at any time after transplantation, n (%) | 10 (33) | 3 (43) | 2 (17) | 5 (45) | *0.337* | *0.305* | *1.000* | *0.193* |
| ·       BKV replication over detection limit, n (%) | 8 (27) | 1 (14) | 2 (17) | 5 (45) | *0.320* | *1.000* | *0.316* | *0.193* |
| ·       BKV positivity below detection limit, n (%) | 2 (7) | 2 (29) | 0 (0) | 0 (0) | *0.048** | *0.123* | *0.137* | *1.000* |
| BKV replication between transplantation and first biopsy, n (%) | 8 (27) | 3 (43) | 1 (8) | 4 (36) | *0.192* | *0.117* | *1.000* | *0.155* |
| BKV replication at time of first biopsy, n (%) | 2 (7) | 1 (14) | 0 (0) | 1 (9) | *0.503* | *0.368* | *1.000* | *0.478* |
| CMV replication at any time after transplantation, n (%) | 23 (77) | 7 (100) | 8 (67) | 8 (73) | *0.269* | *0.245* | *0.245* | *1.000* |
| CMV replication between transplantation and first biopsy, n (%) | 15 (50) | 6 (86) | 4 (33) | 5 (45) | *0.111* | *0.057* | *0.151* | *0.680* |
| CMV replication at time of first biopsy, n (%) | 2 (7) | 1 (14) | 0 (0) | 1 (9) | *0.503* | *0.368* | *1.000* | *0.478* |

*median (range)

**C**

|  | **Total**  n=30 | **Deteriorating MVI "+"**  n=7 | **Stable MVI "0"**  n=12 | **Improving MVI "-"** n=11 | ***P***  *all groups* | ***P***  *+ vs. 0* | ***P***  *+ vs. -* | ***P***  *0 vs. -* |
| --- | --- | --- | --- | --- | --- | --- | --- | --- |
| **2. Renal biopsy** |  |  |  |  |  |  |  |  |
| Time post-1st biopsy, months* | 2 (0-24) | 6 (0-24) | 0.5 (0-19) | 1 (0-17) | *0.241* | *0.120* | *0.211* | *0.695* |
| ·       <6 months, n (%) | 19 (63) | 3 (43) | 9 (75) | 7 (64) | *0.400* | *0.326* | *0.630* | *0.667* |
| ·       6-12 months, n (%) | 5 (17) | 2 (29) | 1 (8) | 2 (18) | *0.596* | *0.523* | *1.000* | *0.590* |
| ·       13-25 months, n (%) | 6 (20) | 2 (29) | 2 (17) | 2 (18) | *0.857* | *0.603* | *1.000* | *1.000* |
| Indication for second biopsy |  |  |  |  |  |  |  |  |
| ·       eGFR, n (%) | 8 (27) | 2 (29) | 4 (33) | 2 (18) | *0.870* | *1.000* | *1.000* | *0.640* |
| ·       Proteinuria, n (%) | 3 (10) | 2 (29) | 0 (0) | 1 (9) | *0.160* | *0.123* | *0.528* | *0.478* |
| ·       eGFR and proteinuria, n (%) | 4 (13) | 0 (0) | 3 (25) | 1 (9) | *0.412* | *0.263* | *1.000* | *0.590* |
| ·       DSA, n (%) | 3 (10) | 0 (0) | 1 (8) | 2 (18) | *0.594* | *1.000* | *0.497* | *0.590* |
| ·       Follow-Up biopsy, n (%) | 12 (40) | 3 (43) | 4 (33) | 5 (45) | *0.894* | *1.000* | *1.000* | *0.680* |
| Banff-Score, n (%) |  |  |  |  |  |  |  |  |
| ·       t-lesion ≥ 2 | 3 (10) | 0 (0) | 2 (17) | 1 (9) | *0.772* | *0.509* | *1.000* | *1.000* |
| ·       t-lesion = 1 | 23 (77) | 7 (100) | 7 (58) | 9 (82) | *0.129* | *0.106* | *0.497* | *0.371* |
| ·       i-lesion ≥ 2 | 1 (3) | 0 (0) | 0 (0) | 1 (9) | *0.600* | *1.000* | *1.000* | *0.478* |
| ·       i-lesion = 1 | 5 (17) | 1 (14) | 2 (17) | 2 (18) | *1.000* | *1.000* | *1.000* | *1.000* |
| ·       ti-lesion ≥ 2 | 8 (27) | 4 (57) | 3 (25) | 1 (9) | *0.087* | *0.326* | *0.047** | *0.590* |
| ·       ti-lesion = 1 | 10 (33) | 2 (29) | 4 (33) | 4 (36) | *1.000* | *1.000* | *1.000* | *1.000* |
| ·       ptc-lesion ≥ 2 | 20 (67) | 6 (86) | 9 (75) | 5 (45) | *0.173* | *1.000* | *0.151* | *0.214* |
| ·       ptc-lesion = 1 | 4 (13) | 1 (14) | 0 (0) | 3 (27) | *0.145* | *0.368* | *1.000* | *0.093* |
| ·       v-lesion ≥ 2 | 1 (3) | 0 (0) | 0 (0) | 1 (9) | *0.600* | *1.000* | *1.000* | *0.478* |
| ·       v-lesion = 1 | 5 (17) | 0 (0) | 4 (33) | 1 (9) | *0.240* | *0.245* | *1.000* | *0.317* |
| ·       cv-lesion ≥ 2 | 4 (13) | 0 (0) | 4 (33) | 0 (0) | *0.061* | *0.245* | *1.000* | *0.093* |
| ·       cv-lesion = 1 | 7 (23) | 2 (29) | 2 (17) | 3 (27) | *0.750* | *0.603* | *1.000* | *0.640* |
| ·       g-lesion ≥ 2 | 15 (50) | 6 (86) | 5 (42) | 4 (36) | *0.135* | *0.147* | *0.066* | *1.000* |
| ·       g-lesion = 1 | 8 (27) | 0 (0) | 4 (33) | 4 (36) | *0.220* | *0.245* | *0.119* | *1.000* |
| ·       cg-lesion ≥ 2 | 12 (40) | 5 (71) | 3 (25) | 4 (36) | *0.163* | *0.074* | *0.335* | *0.667* |
| ·       cg-lesion = 1 | 7 (23) | 1 (14) | 3 (25) | 3 (27) | *1.000* | *1.000* | *1.000* | *1.000* |
| ·       mm-lesion ≥ 2 | 0 (0) | 0 (0) | 0 (0) | 0 (0) | *1.000* | *1.000* | *1.000* | *1.000* |
| ·       mm-lesion = 1 | 4 (13) | 2 (29) | 1 (8) | 1 (9) | *0.514* | *0.523* | *0.528* | *1.000* |
| ·       ci-lesion ≥ 2 | 7 (23) | 2 (29) | 3 (25) | 2 (18) | *1.000* | *1.000* | *1.000* | *1.000* |
| ·       ci-lesion = 1 | 14 (47) | 4 (57) | 4 (33) | 6 (55) | *0.523* | *0.377* | *1.000* | *0.414* |
| ·       ct-lesion ≥ 2 | 7 (23) | 2 (29) | 3 (25) | 2 (18) | *1.000* | *1.000* | *1.000* | *1.000* |
| ·       ct-lesion = 1 | 22 (73) | 5 (71) | 8 (67) | 9 (82) | *0.870* | *1.000* | *1.000* | *0.640* |
| ·       ah-lesion ≥ 2 | 14 (47) | 4 (57) | 5 (42) | 5 (45) | *0.897* | *0.650* | *1.000* | *1.000* |
| ·       ah-lesion = 1 | 14 (47) | 2 (29) | 7 (58) | 5 (45) | *0.468* | *0.350* | *0.637* | *0.684* |
| ·       aah-lesion ≥ 2 | 12 (40) | 3 (43) | 3 (25) | 6 (55) | *0.403* | *0.617* | *1.000* | *0.214* |
| ·       aah-lesion = 1 | 2 (7) | 0 (0) | 2 (17) | 0 (0) | *0.326* | *0.509* | *1.000* | *0.478* |
| ·       cd4-lesion ≥ 2 | 9 (30) | 2 (29) | 5 (42) | 2 (18) | *0.538* | *0.656* | *1.000* | *0.371* |
| ·       cd4-lesion = 1 | 2 (7) | 1 (14) | 0 (0) | 1 (9) | *0.503* | *0.368* | *1.000* | *0.478* |
| **Immunosuppression** |  |  |  |  |  |  |  |  |
| Steroid, n (%) | 16 (53) | 3 (43) | 6 (50) | 7 (64) | *0.721* | *1.000* | *1.000* | *0.680* |
| Privigen, n (%) | 9 (30) | 3 (43) | 4 (33) | 2 (18) | *0.605* | *1.000* | *0.326* | *0.640* |
| Rituximab, n (%) | 3 (10) | 1 (14) | 2 (17) | 0 (0) | *0.431* | *1.000* | *0.389* | *0.478* |
| PEX, n (%) | 6 (20) | 1 (14) | 3 (25) | 2 (18) | *1.000* | *1.000* | *1.000* | *1.000* |
| Other, n (%) | 5 (17) | 1 (14) | 3 (25) | 1 (9) | *0.822* | *1.000* | *1.000* | *0.590* |
| **Kidney transplant function** |  |  |  |  |  |  |  |  |
| Creatinine baseline 1 year before second biopsy, μmol/L* | 165.57 (96.67-347.67) | 144.86 (112.50-180.33) | 189.64 (101.33-347.67) | 148 (96.67-212.00) | *0.496* | *0.404* | *1.000* | *0.310* |
| Creatinine at second biopsy, μmol/L* | 190.31 (97.00-324.00) | 165.86 (100.00-239.00) | 209.75 (113.00-324.00) | 184.65 (97.00-314.50) | *0.515* | *0.261* | *0.724* | *0.525* |
| Creatinine baseline 1 year after second biopsy, μmol/L* | 150.51 (61.00-289.00) | 141.89 (90.00-172.00) | 162.51 (61.00-289.00) | 140.24 (80.67-219.33) | *0.723* | *0.750* | *0.689* | *0.464* |
| Proteinuria baseline 1 year before second biopsy, mg/day* | 425.8 (50.00-1'286.67) | 645.33 (50.00-1'286.67) | 347.67 (100.00-883.33) | 386.25 (63.33-1'050.00) | *0.623* | *0.440* | *0.435* | *1.000* |
| Proteinuria at second biopsy, mg/day* | 2'073.79 (0-37'960.00) | 900 (50.00-1'870.00) | 4'072.50 (0-37'960.00) | 497 (0-1'390.00) | *0.548* | *0.482* | *0.230* | *0.872* |
| Proteinuria baseline 1 year after second biopsy, mg/day* | 519.2 (0-2'176.67) | 929.44 (50.00-2'176.67) | 478.19 (0-1'806.67) | 300.37 (0-783.33) | *0.405* | *0.213* | *0.328* | *0.972* |
| **Viral infections** |  |  |  |  |  |  |  |  |
| BKV replication between first and second biopsy, n (%) | 5 (17) | 1 (14) | 2 (17) | 2 (18) | *1.000* | *1.000* | *1.000* | *1.000* |
| BKV replication at time of second biopsy, n (%) | 2 (7) | 1 (14) | 1 (8) | 0 (0) | *0.697* | *1.000* | *0.389* | *1.000* |
| CMV replication between first and second biopsy, n (%) | 8 (27) | 2 (29) | 4 (33) | 2 (18) | *0.870* | *1.000* | *1.000* | *0.640* |
| CMV replication at time of second biopsy, n (%) | 0 (0) | 0 (0) | 0 (0) | 0 (0) | *1.000* | *1.000* | *1.000* | *1.000* |

*median (range)
